# The role of intestinal microbes on intestinal barrier function and host immunity from a metabolite perspective

**DOI:** 10.3389/fimmu.2023.1277102

**Published:** 2023-10-09

**Authors:** Yifeng Fu, Jin Lyu, Shuangshuang Wang

**Affiliations:** ^1^ Department of Cardiology, The Affiliated Wenling Hospital of Wenzhou Medical University (The First People’s Hospital of Wenling), Wenling, Zhejiang, China; ^2^ College of Bioscience and Biotechnology, Hunan Agricultural University, Changsha, Hunan, China; ^3^ Department of Pathology, the First People’s Hospital of Foshan, Foshan, Guangdong, China

**Keywords:** intestinal microorganisms, short-chain fatty acids, tryptophan, AHR, dietary fibre

## Abstract

The gut is colonized by many commensal microorganisms, and the diversity and metabolic patterns of microorganisms profoundly influence the intestinal health. These microbial imbalances can lead to disorders such as inflammatory bowel disease (IBD). Microorganisms produce byproducts that act as signaling molecules, triggering the immune system in the gut mucosa and controlling inflammation. For example, metabolites like short-chain fatty acids (SCFA) and secondary bile acids can release inflammatory-mediated signals by binding to specific receptors. These metabolites indirectly affect host health and intestinal immunity by interacting with the intestinal epithelial and mucosal immune cells. Moreover, Tryptophan-derived metabolites also play a role in governing the immune response by binding to aromatic hydrocarbon receptors (AHR) located on the intestinal mucosa, enhancing the intestinal epithelial barrier. Dietary-derived indoles, which are synthetic precursors of AHR ligands, work together with SCFA and secondary bile acids to reduce stress on the intestinal epithelium and regulate inflammation. This review highlights the interaction between gut microbial metabolites and the intestinal immune system, as well as the crosstalk of dietary fiber intake in improving the host microbial metabolism and its beneficial effects on the organism.

## 1 Introduction

The intestine is widely recognized as the body’s foremost immune organ, with its role in communicating with food, symbiotic microbial communities, and external pathogens. To facilitate these vital connections, the gut has evolved into a highly dynamic structure with the ability to regulate both innate and adaptive immunity (1). However, changes in diet and lifestyle have caused extraordinary rates of gastrointestinal health issues, increasing the incidence of inflammatory bowel disease (IBD) worldwide (2–4).

The host and intestinal flora maintain a reciprocal link when the organism is stable. However, achieving this balance between the commensal microbiota and mucosal immunity can be a challenging task. The microbiota mainly resides in the colon, upper digestive tract, saliva, and throat (5–7). Moreover, gut microbes play a vital role in initiating immunological activation due to their abundance when compared to human cells. The intestinal epithelium acts as a physical and chemical barrier that protects the intestinal mucosa and surrounding organs from harmful microorganisms. However, prolonged interaction between the microorganisms and the intestine may cause IBD. IBD is a chronic, recurrent inflammatory condition caused by various factors such as heredity and environmental factors. The pathology of IBD, with its fluctuating periods of deterioration and remission (8, 9), presents a significant challenge in developing specific treatments against the disease (10).

There have been several studies confirming the impact of the gut microbes on IBD (11, 12). The interaction between microbial-derived metabolites, the intestinal mucosa, and diet plays a crucial role in preventing and treating inflammatory diseases and promoting a balanced host immunity (13–15). These microbial-derived metabolites, also known as gut microbial metabolites, act as messengers, providing information to the host about the microbiome composition, the presence of pathogens, or other environmental challenges (16). Moreover, recent research has highlighted the significant influence of diet on the composition of the gut microbiota, ultimately affecting host health by regulating intestinal permeability and modulating both the innate and adaptive immune systems (17).

This review aims to detail the interactions between microbial metabolites and the intestinal mucosa, and highlight the implications of these interactions on the human immune system. Specifically, we will examine how dietary habits can promote the production of microbial metabolites, thus preventing intestinal inflammation. Additionally, this article aims to provide insights into basic research in this field.

## 2 Gut microbiota and host immunity

The gut microbiota is a diverse ecosystem comprising various microorganisms, such as bacteria, archaea, phages, eukaryotic viruses, and fungi (18). While bacteria have received significant attention, it is worth noting that fungal communities also play a crucial role in this ecosystem. Although they constitute only around 1% of the human gut, fungi have been found to be actively involved in the development of diseases and can significantly influence the host’s immune response (19). It is interesting to note that more than 1000 bacteria that colonize the gastrointestinal tract belong to the Firmicutes and Bacteroidetes phyla, which make up approximately 90% of the entire microbial community (20). The mechanisms by which the intestinal mucosa adapts to the various flora have not been extensively explored, but it is already highly adaptive. The developmental processes of the immune system are driven by microorganisms, and in turn, the immune system influences the composition of the gut microbiota, and changes in microorganisms indirectly affect host immunity (21, 22). Infants have very little intestinal flora before birth (23), and after birth the microorganisms gradually colonise the intestines due to the influence of the mother and the surrounding environment (24), and ultimately the microorganisms reach a steady state in the host, which affects the health of the host (25–27).

Early in life, specific gut bacteria work in tandem with immune tissue surrounding the intestinal mucosa (28). Failure to form an appropriate microbiota at this stage can weaken the immune system, potentially leading to adverse outcomes later in life. Research has demonstrated that gut microbes can affect the recruitment of immune cells and elicit inflammation, as seen in IBD (29, 30). Thus, the maintenance of intestinal immune homeostasis largely relies on the interactions between the microbiota and the intestinal epithelium. Larsen et al. conducted a study which indicated that treatment with lysozyme from the basophilic *Acremonium alcalophilum* during enteritis suppressed inflammation and reversed inflammation-induced changes in the intestinal microbiota. However, this protection was diminished in mice with depleted microbiota treated with antibiotics, suggesting a dependence on the microbiota for lysozyme’s anti-inflammatory impact (31). Additionally, Wu et al. found that *Lactobacillus reuteri* treatment in TNF (Tumor necrosis factor)-induced intestinal inflammation led to a decrease in TNF production, repaired gut damage by activating the Wnt/β-catenin signaling pathway, and increased intestinal epithelial proliferation and differentiation, thereby strengthening the intestinal mucosal barrier against inflammation (32).

Recent studies in mice have shown that the fatty acid oxidation pathway is enhanced during a 24-hour fasting period, resulting in improved activity of intestinal stem cells (33). Fasting cycles have been found to alleviate intestinal inflammation and increase gut stem cells and probiotics, leading to an improvement in the inflammation-related phenotype of IBD (34). The therapeutic effect of probiotics on intestinal inflammation has been confirmed by multiple studies (35, 36). For instance, Xiang et al. demonstrated in a mouse model of DSS (dextran sodium sulfate)-induced enteritis that treatment with *Bifidobacterium breve* strains H4-2 and H9-3 significantly ameliorated colon length shortening, attenuated inflammatory damage to the colon, and restored the number of mucus-secreting goblet cells (37). Another study investigated the effects of *Bifidobacterium adolescentis* treatment in mice with colitis and found that gavage administration of *B. adolescentis* induced the secretion of anti-inflammatory factors while reducing pro-inflammatory factors, effectively alleviating intestinal inflammation compared to the untreated mice with colitis. Furthermore, 16S rRNA sequencing of mouse feces revealed a decrease in the abundance of harmful pathogens *Akkermansia* and *Escherichia-shigella* in the *B. adolescentis*-treated mice (38).

The interaction between the host and gut microbes is essential for establishing immune tolerance and preventing harmful foreign microbes (39). An essential component of this interaction is the mucus layer produced by intestinal epithelial cells, which acts as a physical barrier separating the intestinal lumen from the underlying tissue (40). This mucus barrier regulates the immunogenicity of intestinal antigens and supports the anti-inflammatory properties of dendritic cells, contributing to immune homeostasis (41). Wimonrat et al. found that gavage administration of Candida resulted in more severe intestinal leakage, higher serum endotoxin levels, and dysbiosis of the intestinal microbiota in mice. Additionally, Candida administration significantly increased serum levels of pro-inflammatory factors IL-6 (Interleukin-6) and TNF-α, and exacerbated intestinal inflammatory damage, which were effectively mitigated by administration of *Lactobacillus rhamnosus* L34 (42). Moreover, a study demonstrated that colonisation of *Escherichia coli* 541-15 in mice with enterocolitis effectively attenuated enterocolitis injury, decreased intestinal permeability, reduced centriole clusters in the lamina propria and epithelium, and reduced the expression of pro-inflammatory markers, lipocalin-2 and myeloperoxidase, as found in the faeces of mice colonised with *E. coli* 541-15. This bacterium was able to prevent colitis through inducing IL-10 (Interleukin-10) production in targeted intestinal epithelial CX3CR1^+^ macrophages (43). Casitas B-lineage lymphoma (c-Cbl) is deficient in bone marrow-derived dendritic cells, mice with dendritic cell-specific deletion of c-Cbl exhibit increased susceptibility to DSS-induced colitis (44). Furthermore, activation of c-Cbl by intestinal fungus leads to enhanced resistance against colitis. The protective effects of commensal fungi can be attributed to c-Cbl-mediated induction of IL-10 production by dendritic cells. The role of Secretory immunoglobulin A (SIgA) in regulating intestinal fungal symbiosis and providing protection to patients with ulcerative colitis has been demonstrated (45). SIgA, an antifungal antibody produced in the gut, functions by encapsulating virulence-associated fungal morphotypes. This protective mechanism helps maintain a balanced fungal community within the intestines and contributes to the overall regulation of intestinal health in patients with ulcerative colitis.

Wang et al. discovered that zearalenone (ZEN), a fungal mycotoxin produced by *Fusarium* and known to cause reproductive immunotoxicity in farm animals while also posing a threat to human health through the food chain. The researchers found that recombinant *Bacillus subtilis* 168-expressing ZEN-degrading enzyme effectively inhibits ZEN. This inhibition leads to an increase in the production of the microbial metabolite called butyrate and a decrease in lipopolysaccharide (LPS) production. As a result, the ZEN-induced intestinal barrier toxicity is counteracted, thereby enhancing the defence mechanisms of the reproductive immunity axis (46). Wu et al. discovered that dietary supplementation of chickens with *Enteromorpha prolifera* (EP) and yeast glycoprotein (YG) not only increased the concentration of short-chain fatty acids but also elevated the abundance of beneficial bacteria in the chicken cecum. Furthermore, the researchers observed an increase in dopamine concentration specifically in the EP+YG-treated group. This finding suggests that EP + YG modulates metabolites associated with neurotransmitters and immune responses, indicating a potential role in enhancing both neurological and immunological functions (47).

In conclusion, intestinal microorganisms are able to have a positive impact by interacting with the intestinal mucosa as well as intestinal immune cells, with probiotics strengthening the host’s intestinal immune function, while harmful pathogens enter the surrounding tissues or bloodstream through the leaky gut and have a negative impact on the organism.

## 3 Gut microbial metabolites

**Figure 1:**
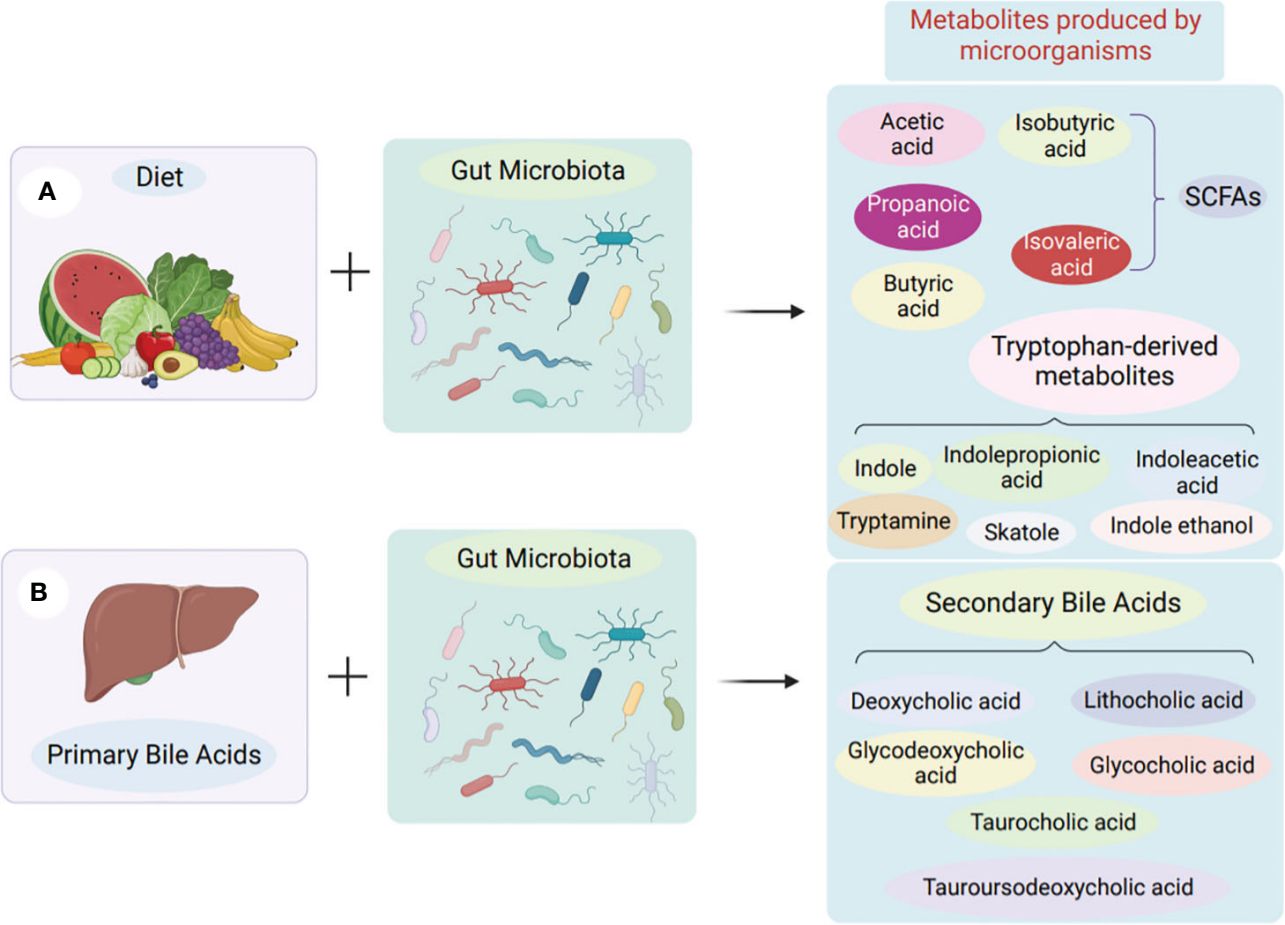
Production of microbial metabolites in the gut. **(A)** Obtained from food. Microorganisms in the colon and cecum produce short-chain fatty acids by fermentation of undigested dietary fibre. Tryptophan-derived metabolites are derived from the direct conversion of tryptophan by gut microbes. **(B)** Synthesis of intestinal bacteria from scratch. Primary bile acids are produced in the liver, transported to the gut and then produced as secondary bile acids by the action of gut microbes.

Gut microbe-derived metabolites play a crucial role as mediators in affecting the growth and operation of the immune system. Additionally, these metabolites interact with intestinal immune cells to mediate immune homeostasis in the gut (48). Small molecule metabolites of a microbial origin have been discovered over the past ten years. These metabolites can be categorized into three main categories: Second, there are metabolites produced by the diet. Third, there are metabolites that are produced by the body and then modified by intestinal bacteria. Finally, there are metabolites that are formed autonomously by intestinal bacteria (see **Figure 1**) (49).

### 3.1 Short-chain fatty acid

#### 3.1.1 Dietary fiber catabolism

Dietary fiber can be partially broken down by microorganisms in the large intestine but is not digested and absorbed in the small intestine. In the complex ecosystem of the gut, these microorganisms convert the sugars in food into metabolites that can have different health effects (50). The human digestive tract lacks the enzymes necessary to break down dietary fiber and polysaccharides, so it relies on specific bacteria in the colon to perform this task. The main bacterial groups responsible for breaking down dietary fiber are the thick-walled and actinomycete phyla, and only a few enzymes are involved in initiating the degradation process (51).

The definition of dietary fiber, as delineated by the Codex Alimentarius, characterizes it as a polymer derived from natural carbohydrates inherent in cereals and fruits or acquired through physicochemical processes involving raw materials (52, 53). A distinctive feature of dietary fiber lies in its solubility, playing a pivotal role in the formation of intestinal gels. Conversely, cellulose, hemicellulose, and lignin are classified as insoluble dietary fibers. For example, many soluble dietary fibers (pectin and guar gum) can be found in plant cell walls. They are the main source of energy for the microorganisms of the gastrointestinal tract. Numerous soluble dietary fibers, such as pectin and guar gum, are present within plant cell walls.

**Figure 2:**
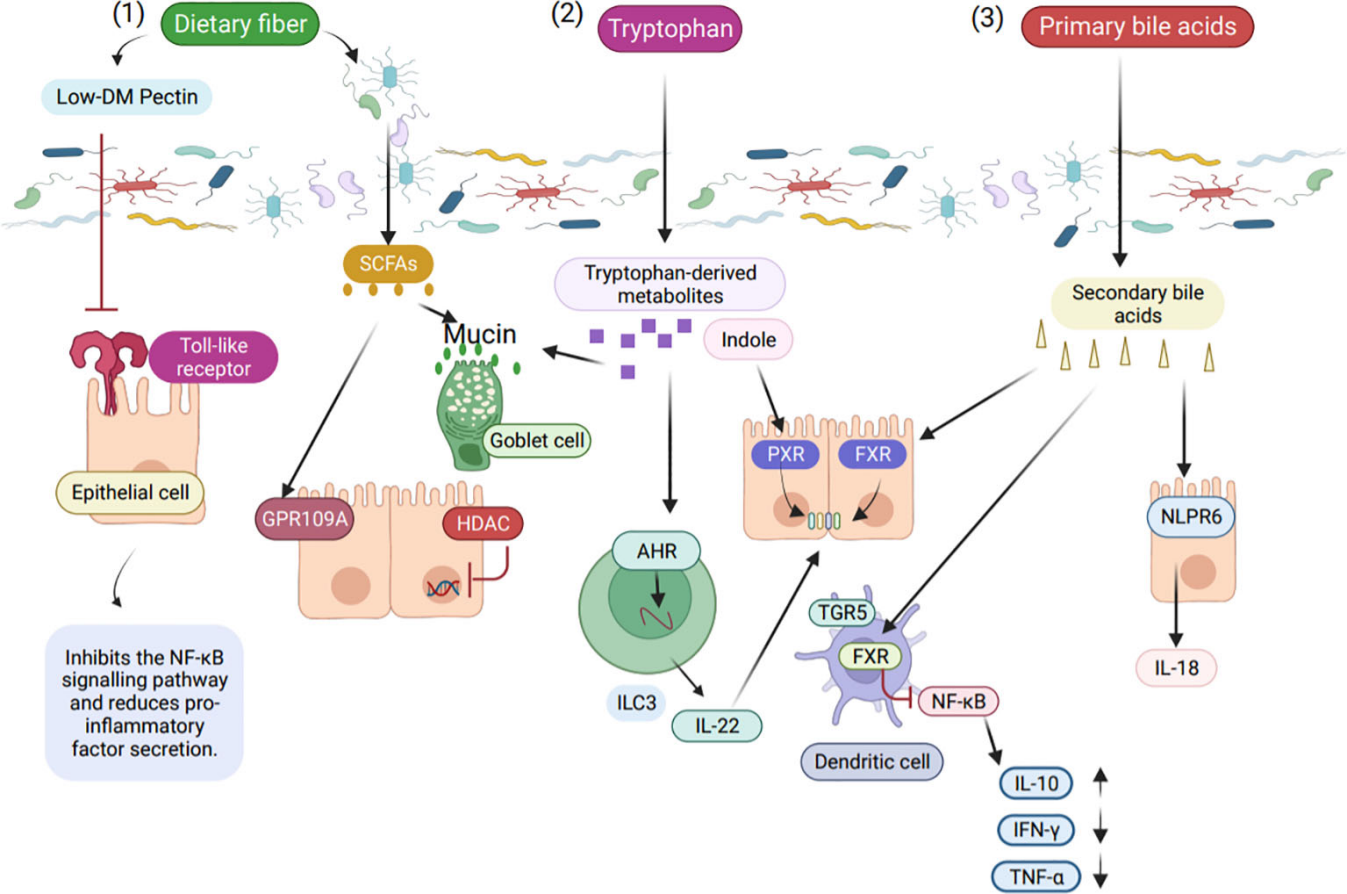
The role of intestinal microbial metabolites in the intestinal immune barrier. (1) Low ester pectin, a product of dietary fiber, binds to Toll like receptor 2 (TLR2) and suppresses TLR2 receptor activation, hence lowering NF-κB (Nuclear transcription factor-κB) activity. SCFA are produced by gut microbes in response to dietary fiber stimulation, and these fatty acids stop histone deacetylases and pro-inflammatory mediators caused by NF-κB. (2) Intestinal microbial-derived tryptophan metabolites, indoles maintain normal intestinal epithelial function via the pregnane X receptor (PXR), and tryptophan metabolites are AHR ligands in innate lymphocytes that act with the AHR via transcription factors to stimulate IL-22 (Interleukin-22) expression. Antimicrobial peptides promote expression through IL-22 and enhance mucin proliferation in goblet cells. (3) Bacterial bile acid metabolites affect intestinal immunity in different ways, with secondary bile acids binding to FXR (Farnesoid X receptor) to protect intestinal integrity.

Dietary fibre intake affects the composition of microorganisms to some extent, which is related to the type of food consumed, and this change affects the production of metabolites by microorganisms (54). Meals that have a high carbohydrate content but low dietary fiber content are linked to a higher risk of inflammation. On the other hand, people on a predominantly high dietary fiber diet have a much a lower risk of developing inflammatory diseases (55). Furthermore, it has been demonstrated that dietary fiber has a positive impact on maintaining the immune system of the intestine. The protective effect of dietary fiber is mainly attributed to the short-chaini-inflammatory effects by inhibit fatty acids (SCFA) produced by fermentation, which are known to act on activating or inhibiting inflammation (**Figure 2**) (52). It is important to note that inflammation and autoimmune illness can result from dysfunction of the intestinal barrier (56).

SCFA are created when components like inulin and wheat are fermented by bacteria, and resistant starch is the main raw material for butyrate production (10). Bacteroidetes create acetate and propionate, while Firmicutes control the formation of butyrate. Lactic acid is produced by *Bifidobacterium*, a type of actinomyces, during the breakdown of dietary fiber. Butyrate, an anti-inflammatory substance, has a powerful ability to reduce the production of pro-inflammatory cytokines (57). Additionally, butyrate enhances intestinal integrity and barrier function by inducing the relocation of ZO-1 (Zonula Occludens-1) and occludin in the cell membrane and increasing the expression of claudin-1 (tight junction protein 1) (58).

#### 3.1.2 SCFA and receptors

Of the SCFA produced by the fermentation of dietary fiber, acetate, propionate, and butyrate are the predominant SCFA, accounting for approximately 95% of the total SCFA concentration (59). These SCFA serve as a source of energy for colon cells and also play a crucial role in regulating cholesterol synthesis and glucose metabolism (60). Moreover, SCFA have been found to improve intestinal barrier function through various mechanisms such as inhibiting pathogen development, reducing intestinal inflammation, and modulating the structure of Tight junctions (TJ) (61, 62). SCFA facilitates the formation of TJ proteins and enhances the intestinal epithelial barrier function (63).

SCFA is detected by a broad collection of human genes that encode protein receptors called GPCRs (G protein-coupled receptors). These receptors include GPR41 (FFAR3), GPR42, GPR43 (FFAR2), GPR109A (HCAR2), GPR164 (OR51E1), and OR51E2 (64). The GPCR family of receptors inhibits the activation of NF-κB in immune cells and intestinal epithelial cells (65). Among the GPCRs, FFAR2 receptors are involved in the β-inhibitor-protein-2 mediated signalling pathway and generate anti-inflammatory effects by inhibiting NF-κB (66). In addition, GPR41 and GPR43 play a crucial role in monitoring immunity to microorganisms in the intestinal mucosa, while GPR109A, which is a tumor suppressor, inhibits the activation of NF-κB (67). TNF-α levels or a reduction in the presence of butyric acid-producing bacteria could be the result of the downregulation of MCT1 (Monocarboxylate transporter 1) expression in the mucosa of patients with ulcerative colitis (68). This indicates that butyric acid reduces GPR109A-mediated expression of IL-8 (Interleukin-8) (69). Moreover, GPR43 regulates immune cells, and NLRP3 (NOD-like receptor thermal protein domain associated protein 3) inflammatory vesicles are activated through GPR41 and GPR43, resulting in IL-1β (interleukin-1β) and IL-18 (interleukin-18) secretion that influences inflammation (70).

Neutrophils, which are a type of immune cell, are known for their high levels of FFAR2 expression (71). In the context of IBD, neutrophils play a role in its pathogenesis. They can migrate to the lamina propria and epithelium to eliminate antigens, thus making a contribution to intestinal homeostasis and the recovery from IBD (10). The maintenance of immune homeostasis requires the regulation of FFAR2, which is strongly expressed in colonic epithelial cells and T regulatory (Treg) cells, by SCFA (72).

The role of SCFA in regulating glucose metabolism disorders is also noteworthy. A study conducted on diabetic patients revealed that long-term infusion of propionate into the colon was successful in improving the weight of overweight adults and preventing complications associated with insulin resistance (73). Furthermore, the GPR43-mediated AMP-activated protein kinase (AMPK) signaling pathway was found to increase AKT (phosphokinase B) phosphorylation in specific hepatocytes, thus influencing diabetes management (74).

The main source of energy is butyrate, which controls inflammation and gene expression (50). A study has shown that butyrate is beneficial to the lumen of the intestine at low concentrations, while too high concentrations can damage the intestinal barrier (75). Excessive concentrations of SCFA have been found to cause mucosal damage in rats, but this damage disappears with the maturation of the mucosa (76). It has been proposed that high butyrate concentrations are harmful for the formation of TJ and the intestinal barrier (77), whereas low butyrate concentrations in Caco-2 cells (human colorectal adenocarcinoma cells) make it easier for TJ to form during the AMPK-mediated processes (62). The mechanism by which butyrate affects inflammation involves histone acetylation (78). Butyrate is the most effective inhibitor of histone deacetylase (HDAC), which also promotes the transcription of specific genes that support intestinal homeostasis in the colon (79). Butyrate is taken up by MCT1 and SMCT1 (Sodium-coupled monocarboxylate transporter 1) at the top of the intestinal epithelium or immune cells through non-ionic diffusion (80).

SCFA mediate the inflammatory processes by interacting with receptors. However, the effects of butyrate on mucosal homeostasis can vary and are subject to context-dependent regulation. For example, low concentrations of butyrate promote the formation of TJ through AMPK-mediated processes. Conversely, studies have shown high levels of butyrate can damage the intestinal barrier, possibly by inhibiting the formation of tight junctions. Additionally, it has been found that butyrate can regulate gene expression by inhibiting HDAC. Overall, the interaction between SCFA and mucosal immunity is complex and deserves further investigation to gain insights into health and disease.

### 3.2 Bile acids

#### 3.2.1 Bile acid metabolism

Bile acids (BA) are steroid molecules produced by cholesterol in liver cells (81). These BA molecules are transported in the small intestine and undergo uncoupling by microbial bile salt hydrolase (BSH). Once uncoupled, the BA is reabsorbed in the ileum through an apical sodium-dependent BA transporter protein (82). The conversion of primary BA to secondary BA primarily occurs through the 7α-hydroxylation reaction, which is catalyzed by bacteria like Clostridium perfringens and eubacteria (83, 84).

**Figure 3:**
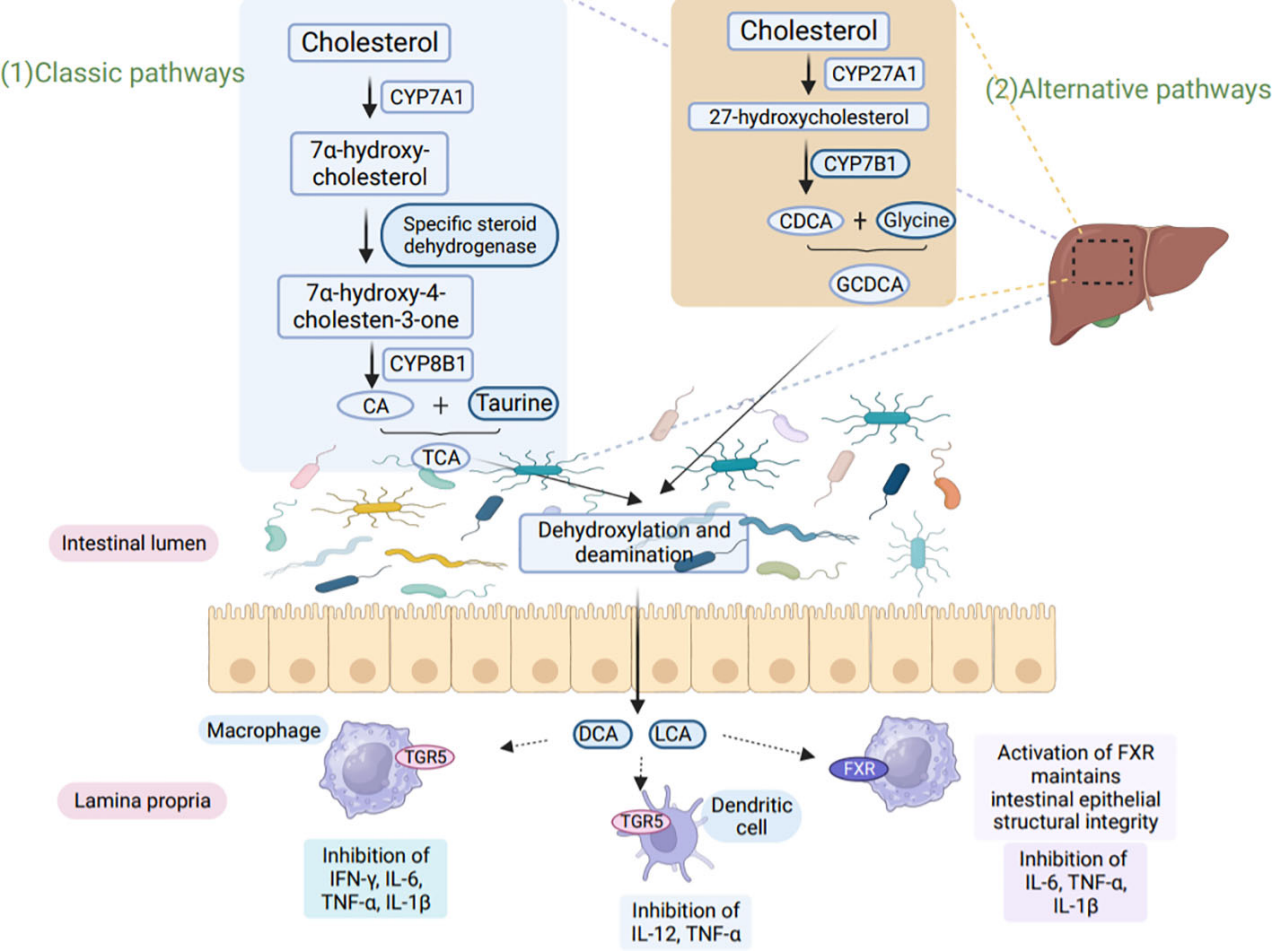
BA metabolism affects host immunity. (1) In the classical pathway, CYP7A1 converts cholesterol to 7α-hydroxy-cholesterol, which is then further converted to hydroxy-4-cholesten-3-one through the action of Steroid dehydrogenase. Another enzyme, CYP8B1, hydroxylates hydroxy-4-cholesten-3-one to produce CA. CA binds to taurine, resulting in the formation of TCA. (2) In the alternative pathway, cholesterol is converted to 27-hydroxycholesterol by CYP27A1, followed by the production of CDCA through the action of CYP7B1. CDCA then binds to glycine to form GCDCA. Both TCA and GCDCA are transported through the bile to the intestine, where they undergo dehydroxylation and deamination by microorganisms. This microbial action leads to the production of secondary bile acids, LCA, and DCA. LCA and DCA interact with TGR5 and FXR present on macrophages and dendritic cells, leading to the inhibition of pro-inflammatory factor secretion. Furthermore, these bile acids play a crucial role in maintaining intestinal epithelial barrier function and exert immunoprotective effects. CYP8B1, sterol 12α-hydroxylase; CA, cholic acid; CDCA, chenodeoxycholic acid; TCA, taurocholic acid; GCDCA, glycochenodeoxycholic acid; LCA, lithocholic acid; DCA, deoxycholic acid.

BA metabolism involves two main pathways, the ‘classical’ and ‘alternative’ pathways (85). In the ‘classical’ pathway, cholesterol-7α-hydroxylase (CYP7A1) converts cholesterol to 7α-OH-cholesterol. In the ‘alternative’ pathway, cholesterol is hydroxylated by sterol 27 hydroxylase (CYP27A1) (86). The main mode of microbial BA conversion in humans is the conversion of primary BA to secondary BA via 7α-hydroxylation reactions (87). These pathways ultimately produce two primary BAs: bile acid and Chenodeoxycholic acid (88). BA and Chenodeoxycholic acid further form conjugated bile salts by combining with glycine or taurine (89). Ultimately BAs affect host immunity, as shown in **Figure 3** (90).

BA bound to glycine or taurine is amphiphilic, meaning that it has both hydrophilic and hydrophobic regions. This quality helps to increase the susceptibility of dietary triglycerides to lipase and thereby promotes efficient fat absorption in the small intestine (91). Although a minor portion of the BA enters the colon, where it is digested and regulated by intestinal microbes for BA production, the majority of the BA is absorbed in the ileum and circulates to the liver (92). Importantly, the daily BA production in a healthy human body is diet-dependent, and BA levels normally remain stable between 200-600 mg (93). Despite this, the link between BA and certain diseases has led to increased attention on gut microbe-mediated BA metabolism (94).

Many bacteria play a role in the uncoupling of BA, but BSH-encoding species are limited to *lactobacillus*, *bifidobacterium*, *Bacteroides*, and *clostridium* (95). The abundance of BSH in gram-positive bacteria is particularly noteworthy. The close correlation between gut microbial BA metabolism and gastrointestinal health has gained significant research attention. Therefore, scholarly interest to investigate the connection between microbes and BA will contribute to a better understanding of liver and colonic diseases.

#### 3.2.2 Secondary BA and receptors

FXR, the vitamin D receptor (VDR), and PXR are powerful secondary BA receptors that can bind secondary BA. The activation of microbial G protein-coupled BA receptors 1 (TGR5) by secondary BA has been found to be involved in the regulation of BA synthesis and metabolism (96). Although TGR5 is expressed in a variety of cells, it is mainly found in macrophages and monocytes (97, 98), and it is activated by bacterial antigens (99). In the absence of the BA-activated receptors FXR, TGR5, PXR, and VDR, the intestinal barrier becomes compromised and disturbed, allowing the translocation of bacteria (100). Thus, investigating the interplay between the BA-activated receptors and bacteria may provide insights into intestinal permeability and dysfunction, as well as immunological and metabolic diseases.

The BA metabolism of FXR involves the inhibitory effect of BA synthesis inhibition, which is mediated by CYP7A1. This step takes place in the hepatocellular bile salt export pump and induces small heterodimeric chaperone (SHP) expression (101). SHP inactivates liver homologous receptor-1 (LRH-1), which represses CYP7A1 expression. Additionally, LRH-1 can inhibit CYP7A1 expression (102). Thus, the interaction between these molecules plays a crucial role in BA metabolism. Moreover, gut immune responses driven by gut microbes are modulated by FXR in response to inflammation. These immune responses may be associated with dysbiosis or dysregulation of BA metabolism (103). Further investigation into the relationship between bile acid metabolism and gut immune responses may provide insights into the mechanisms underlying these complex processes.

It has been shown that PXR acts as a sensor for LCA and can reduce the gene expression of LCA to minimize damage to the host, which is necessary to balance the intestinal barrier and inflammatory homeostasis (104). Patients suffering from intestinal inflammation exhibit lower levels of bile salts as well as lower secondary and higher BA levels in the organism compared to normal subjects (105). To further understand the impact of secondary BA deficiency on enterocolitis, experiments conducted by Sidhartha R et al. demonstrated that patients with ulcerative colitis have significantly lower expression of BA-inducible genes, these genes are responsible for the critical 7α-hydroxylation reaction, which converts primary BA to secondary BA (106). Additionally, Wang et al. conducted a study on the effect of a high-fat diet (HFD) on colitis in wild-type mice. They found that the HFD increased levels of goose deoxycholic acid, leading to macrophage activation and the initiation of colonic inflammation (107). In a mouse model of colitis, the mRNA expression of FXR, a key regulator of BA metabolism, was found to be downregulated in the intestinal mucosa of mice with an inflammatory phenotype. This demonstrates a potential association between FXR and the onset of IBD (99). Moreover, the deletion of FXR in mice prevents remission of enteritis due to the accumulation of inflammatory cells, as well as the stimulatory effect of NF-κB on intestinal microbial-lipopolysaccharide (108).

Gut microbes can activate TGR5, which impacts the expression of enteroendocrine cells involved in immune regulation and anti-inflammation (109). This, in turn, directly influences macrophage polarization and the subsequent inflammatory response. Activation of TGR5 also leads to the production of the hormone glucagon-like peptide-1 (GLP-1) and controls glucose metabolism. Once TGR5 is activated, BA suppress the production of inflammatory cytokines such as IL-1 (Interleukin-1), IL-6, and TNF-α (110). The pro-inflammatory properties of TGR5 can be modulated by BSH-containing bacteria that cause the dissociation of taurine or glycine from BA (52).Additionally, both vitamin D deficiency and down-regulation of VDR expression are risk factors for the increased incidence of intestinal inflammation (111).

### 3.3 Tryptophan-derived metabolites

#### 3.3.1 Tryptophan catabolism

Tryptophan, an essential amino acid, is naturally found in a variety of foods including poultry, milk, tuna, fish, cheese, bread, oats, plums, chocolate, and peanuts. Upon ingestion, tryptophan undergoes catabolism through three distinct pathways: the indole pathway, created by microorganisms in the intestine; the 5-hydroxytryptamine pathway, produced by chromophores in the intestine; and the kynurenine pathway, produced by immune cells and the intestinal mucosa (112).

In mammalian cells, the kynurenine pathway is initiated via tryptophan-2,3-dioxygenase (TDO) and indoleamine 2,3-dioxygenase (IDO1) (113). IDO1 is abundantly expressed in the gut and is connected to immune control, making it the most important tryptophan metabolizing enzyme for immune function (114). IDO1 activation is induced by the body’s inflammatory response and the release of inflammatory cytokines (115). This activation acts to prevent excessive inflammatory responses (116). Upon initiating the kynurenine synthesis pathway, IDO1 and TDO produce formylkynurenine, which is then converted to kynurenine by kynurenine formamidase (112). Kynurenine itself can be further transformed by kynurenine transaminase into kynurenic acid, by kynureninase into anthranilic acid, or by kynurenine monooxygenase into 3-hydroxykynurenine (3-HK) (112).

**Figure 4:**
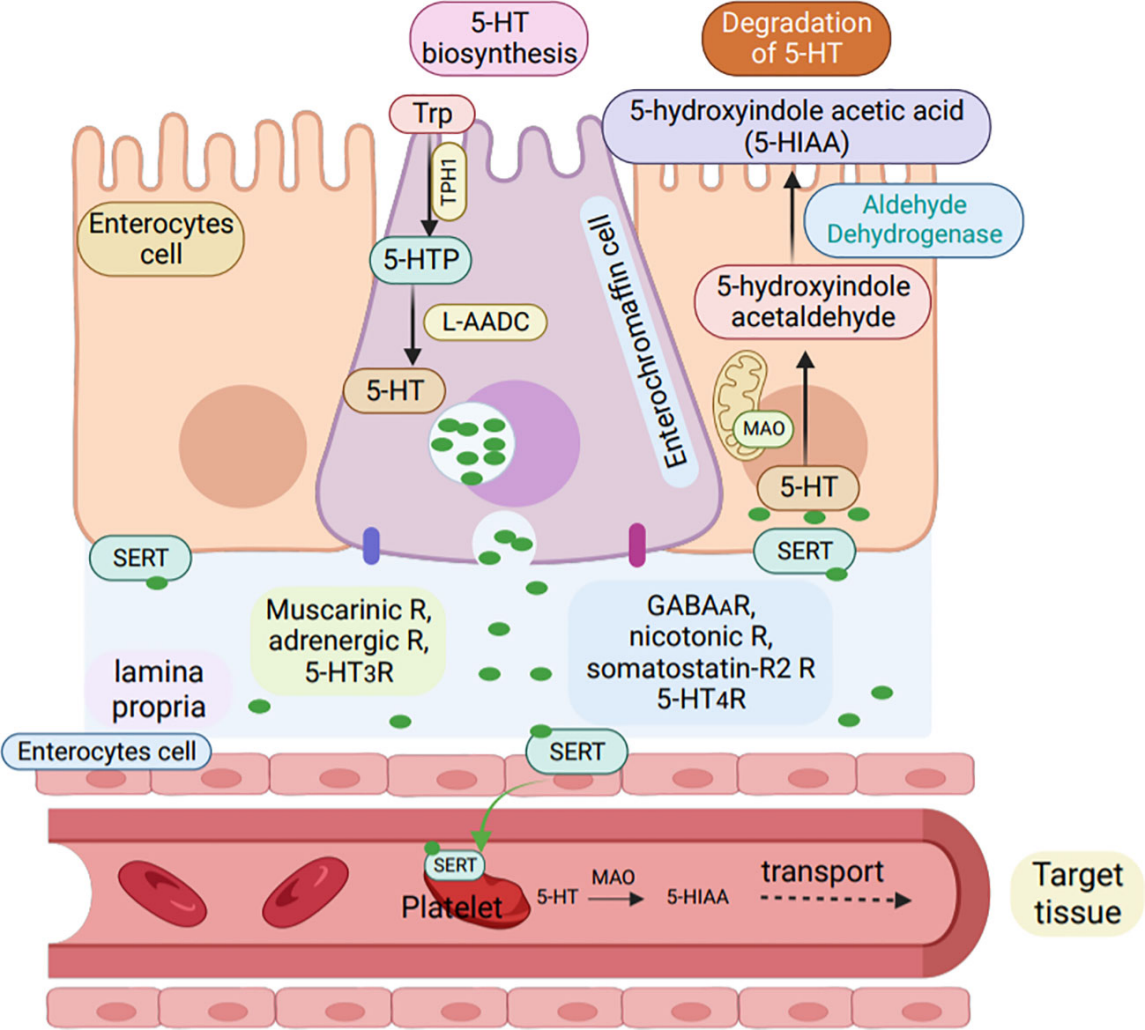
Synthesis and degradation of serotonin in enterochromaffin cells. Serotonin (5-HT) is synthesized by enterochromaffin cells (purple) in the GI tract from L-tryptophan via the rate-limiting enzyme TPH-1.L-5-hydroxytryptophan is then converted into active 5-HT by L-aromatic acid decarboxylase (L-AADC) and stored in enterochromaffin granules. Apically, enterochromaffin cells are stimulated to secrete 5-HT by GPCR in the colon and by glucose-dependent in-sulinotropic peptide-1 in the small intestine, while 5-HT4R inhibits 5-HT release. Basolaterally, EC cells express muscarinic,adrenergic, and 5-HT3 receptors, activation of which leads to 5-HT release, while activation of GABAA, nicotinic, somatostatin-R2,and 5-HT4R inhibit 5-HT release. enterochromaffin cells or enterocytes (orange) can uptake 5-HT via the serotonin reuptake transporter (SERT)and degrade 5-HT to 5-hydroxindole acetic acid via enzyme MAO (R, receptors).

Impaired canine urine pathways have been found to be associated with immune disorders (115). The conversion of tryptophan to serotonin is catalyzed by the enzyme tryptophan hydroxylase (TPH), followed by the conversion of 5-hydroxytryptophan to serotonin by 5-hydroxytryptophan decarboxylase, the specific mechanism is shown in the **Figure 4** (117). Monoamine oxidase (MAO) then changes serotonin to 5-hydroxyindoleacetaldehyde, which is further converted to 5-hydroxyindoleacetic acid (118).

The gut microbiota can convert tryptophan into various metabolites, including indole, tryptamine, indole ethanol, indole propionic acid, indole lactic acid, indole acetic acid, faecal odorant, indole aldehyde, and indole acrylic acid (119). The production of these metabolites is dependent on the presence of specific catalytic enzymes unique to different bacterial species. Bacteria interact with each other to generate these metabolites, as demonstrated in **Table 1** (135, 138).

**Table 1:** Intestinal microflora metabolites and host effects.

Family	Metabolites	Diet	Effects	References
*Rumenococcus* *Rosebacter Shiba*	Acetate	Foods containing fibre	•Signalling molecules •Source of energy for colon cells	(25, 120)
*Megasphaera elsdenii*, *Veillonella spp*	Propionate	Foods containing fibre	• Immunomodulation • Maintenance of vascular function	(121, 122)
*E. hallii, Eubacterium rectale*	Butyrate	Foods containing fibre	• Regulation of immune cell function	(123, 124)
*Eubacterium* *Fusobacterium*	BA	Solid alcoholic foods	•Promotes lipid absorption •Surfactants	(125, 126)
*Escherichia coli* *Proteus*	Tryptamine	High protein foods	•Inflammatory regulators	(127, 128)
*Escherichia coli, Paracolobactrum coliforme*	Indole	Fibre-rich foods	• Immunomodulation • Signaling molecule	(113, 128)
*Clostridium* *Peptostreptococcus*	Indolepropionic acid (IPA)	Fibre-rich foods	• Treatment of metabolic disorders	(127)
*Clostridium* *Bacteroides*	Indoleacetic acid (IAA)	Dietary tryptophan	• Regulates intestinal homeostasis • Suppressing Inflammation	(129–131)
*Lactobacillus*, *Leuconostoc, and* *Weissella*	Branched-chain amino acids (BCAA)	Regular diet	• Signalling molecules	(132, 133)
*Salmonella*	LPS	Western-style eating	• Inflammatory activation related	(134)
*Pseudomonas* *fluorescens*	‘Kynurenines’ (kynurenine and its derivatives)	Regular diet	• Involvement in the immune response • Regulates the gastrointestinal tract	(135, 136)
*Turicibacter spp*	Serotonin	Dietary intake	• Promotes energy absorption and storage	(135, 137)

Bacteria such as *Enterococcus faecalis* and *E. coli* have the ability to convert tryptophan into indole, which is essential for biofilm formation and can also regulate bacterial motility. Additionally, these bacteria can generate resistance to non-indole-producing species (139). In the intestines, the conversion of tryptophan by intestinal bacteria results in the production of tryptamine, indole pyruvic acid, indole-3-glyoxylic acid, and indole-3-lactic acid (140). Specifically, indole can be produced by thick-walled phyla like *Enterobacter aerogenes* and *E. coli*, as well as some members of the phylum *Bacteroidetes (* 52).

**Figure 5:**
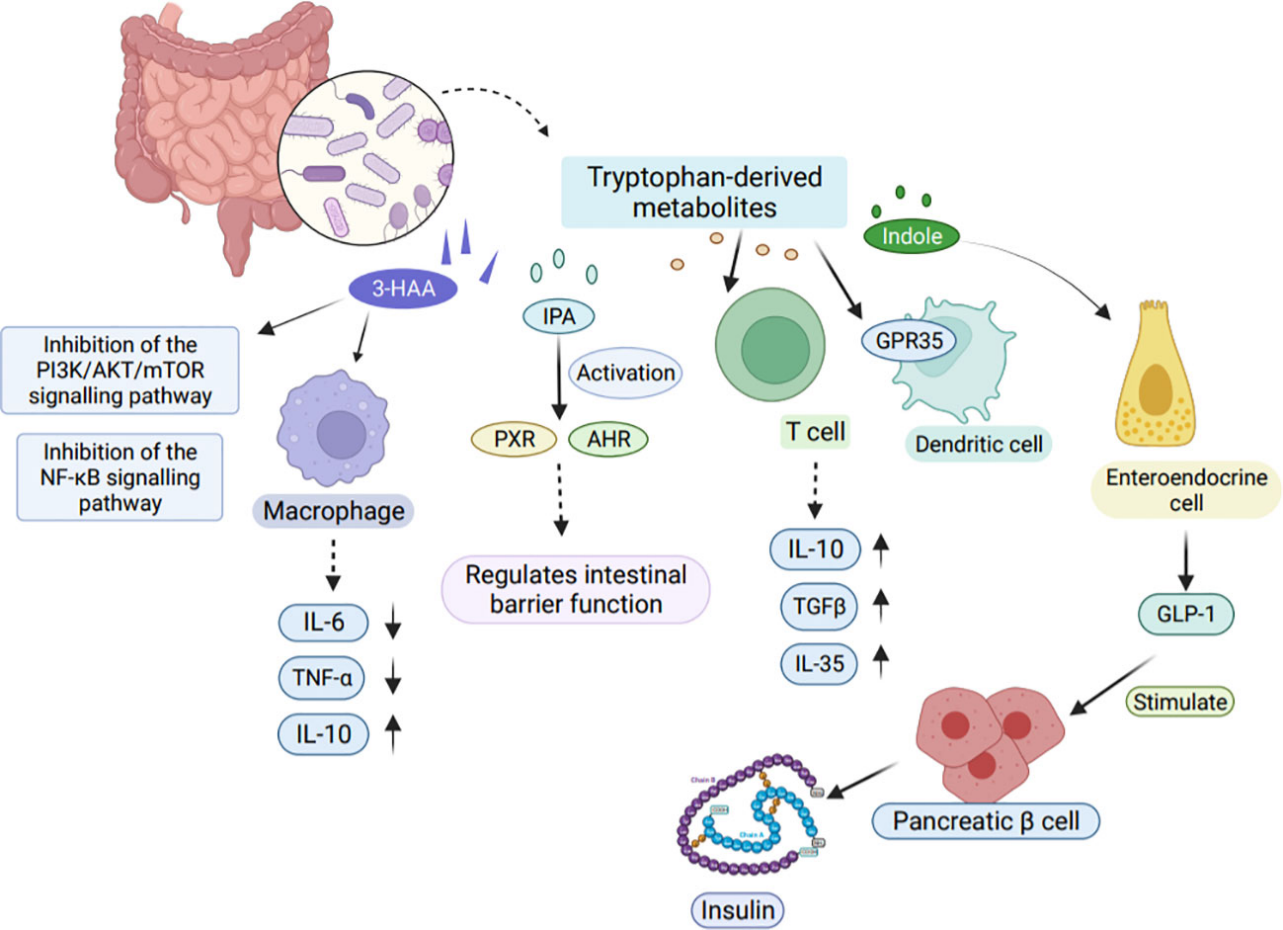
Effects of tryptophan-derived metabolites on host immunity. Tryptophan-derived metabolites can be produced through the direct conversion of tryptophan by gut microbes. One such metabolite, 3-HAA, inhibits the PI3K/AKT/mTOR and NF-κB signaling pathways, leading to reduced production of pro-inflammatory factors IL-6 and TNF-α in macrophages. Another metabolite, IPA, activates PXR and AHR, playing a crucial role in regulating intestinal barrier function. Moreover, tryptophan-derived metabolites also impact T cells by releasing TGFβ, IL-10, and IL-35, which contribute to the suppression of tissue inflammation. Indole, another metabolite, modulates GLP-1 secretion in colonic enteroendocrine cells, thereby stimulating insulin secretion from pancreatic β cells. 3-HAA, 3-Hydroxyanthranilic acid. TGFβ, Transforming growth factor-β.

Tryptophan metabolites produced by bacteria show limited affinity for AHR. Among them, indole, fecal odorant, tryptamine, indolepropionic acid (IPA), and indole-3-acetamide have the highest affinity (141). Reduced dietary intake of tryptophan leads an increased susceptibility of mice to adverse effects of inflammation induction (142). Furthermore, Tryptophan also exhibits regulatory functions in organ development, neurophysiology, and metabolic disorders (143). Tryptophan metabolites play an important role in host immunoprotection as shown in **Figure 5** (144).

#### 3.3.2 Indoles and AHR

The tryptophan-AHR route is a mechanism in which indole can bind to and activate AHR. AHR is a ligand-activated transcription factor that functions as a receptor for many environmental toxins in the immune system (145).

**Figure 6:**
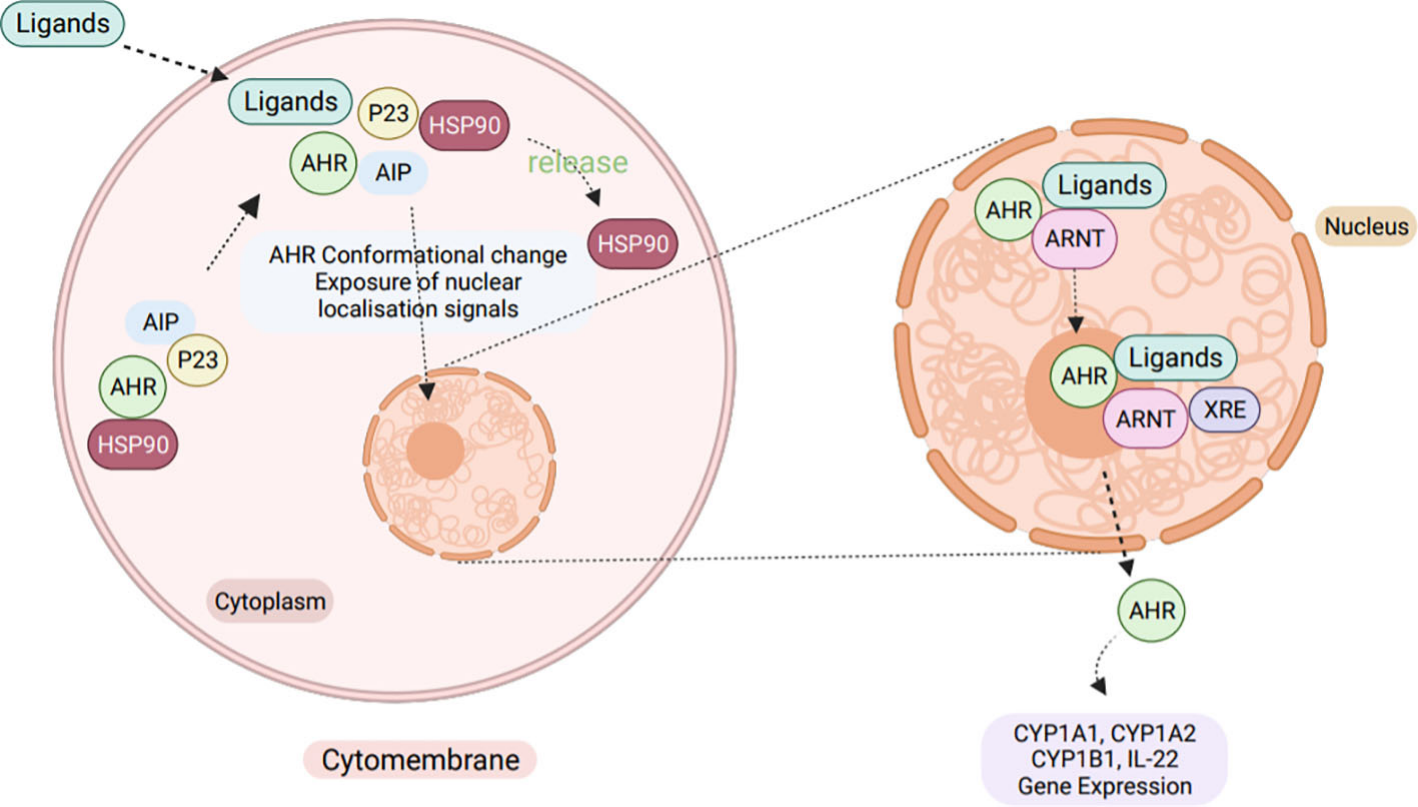
The cellular AHR signaling pathway. Normally, AHR exists in a dormant state within the cytoplasm, bound to a complex of HSP90, XAP2 (X-Associated Protein-2, also known as ARA9 and AIP), and HSP90 Co-chaperonep23. However, upon ligand binding, AHR undergoes a conformational change that exposes a NLS, leading to the release of HSP90 from the complex. This allows the activated AHR to translocate into the nucleus, where it forms a hetero-dimer with the ARNT. Together, the AHR-ARNT heterodimer binds to the XRE of target genes, inducing the expression of genes like IL-22, CYP1A1, CYP1A2, and CYP1B1.

AHR plays a crucial role in immunity by interacting with various regulatory and signaling proteins, such as PAS heterodimerization partners, AHR nuclear translocator (ARNT), and chaperone and immunophilin-like proteins, including Heat Shock Protein-90 (HSP90) and AHR-Interacting Protein p23 (AIP) (146). Upon binding with ligands in the cytoplasm, AHR undergoes a conformational change which results in the exposure of a nuclear localization signal (NLS). This change leads to the release of HSP90 from the complex and allows the receptor to translocate to the nucleus, where it forms a heterodimer with ARNT (147). This activated heterodimer then binds to the xenobiotic response element (XRE) and alters expression of genes controlled by enhancer XREs. Once in the nucleus, AHR quickly forms a heterodimer with ARNT and jointly regulates the expression of downstream target genes, such as the drug metabolizing enzyme cytochrome oxidase 450 1A1 (CYP1A1) (**Figure 6**) (145).

Tryptophan is produced through several metabolic pathways and acts as a ligand for AHR. When these ligands bind to AHR, they can activate downstream target gene expression, such as the expression of IL-22 and IL-17 (Interleukin-17) (112).

The AHR is a vital regulatory protein that interacts with various chaperone and immunophilin-like proteins to carry out its activity (145). Once bound to a ligand, changes in the NLS activate the AHR, prompting it to enter the nucleus. There, the activated AHR binds to ARNT to form a heterodimer that controls the expression of downstream target genes (148). Studies conducted in mice have shown that macrophages from AHR-deficient animals express lower levels of IL-10, while macrophages from AHR-overexpressing mice produce much higher levels of IL-10 (149).

Activation of the tryptophan-AHR pathway is essential for maintaining normal function of the intestinal mucosal barrier. In colitis induced by DSS, expression of key TJ proteins, such as ZO-1, claudin-1, and occludin, is dramatically reduced. However, treatment with the endogenous AHR ligand, 6-formylindolo[3,2-b]carbazole (FICZ), significantly elevates the expression of these TJ proteins (150). FICZ is a tryptophan photochemical product that activates AHR and promotes the synthesis of transforming growth factor (TGF), IL-6, and IL-23 (Interleukin-23). These cytokines enhance the release of IL-22, which plays a critical role in reducing inflammation and differentiation of Th17 cells (145). Inflammatory mediators, such as interferon (IFN) and TNF-α, which are linked to the etiology of IBD, have an impact on the function of TJ (151). However, activation of the tryptophan-AHR pathway prevents the activation of myosin light chain kinase (MLCK) and phosphorylated MLC (pMLC) signaling pathways, leading to the elimination of TNF-α/IFN-γ induced barrier dysfunction in the intestinal mucosa (152).

The study by M. Jennis et al. showed that IPA improved the impaired barrier function of monolayer intestinal epithelial cells in humans (153). Additionally, it was found that IPA positively influences the intestinal barrier, inflammatory response, and differentiation of goblet cells in rodents (154). More recently, Canaan M et al. conducted a study on mice with enteropathy, where they found that administration of indole and indomethacin reduced intestinal damage and maintained normal crypt depth and submucosal thickness (155). Furthermore, they observed that the administration of indole with indomethacin reduced the infiltration of neutrophils and prevented the destruction of tryptophan metabolites, resulting in attenuated changes in the pro-inflammatory mucosal transcriptome. It has been shown that bacteria deficient in metabolizing tryptophan affect the immune regulation of IL-22, and the production of IL-22 in patients with IBD is reduced. In studies of inflammatory patients, changes affecting the IL-22 production pathway have been identified in mice lacking the Card9 gene sensing C-type lectins, making them prone to colitis (156).

The above evidence suggests that the combination of indole and AHR affects the immune process by activating target gene expression and regulating inflammatory factors.

## 4 Conclusion

Gut microbes have been extensively studied and have been shown to play a pivotal role in influencing gut health and organismal immunity. To prevent and treat intestinal diseases more effectively, it is essential to have a thorough understanding of the interactions among gut microbial metabolites, the interacting receptors, and transcriptional regulatory metabolites. The production of SCFA generated by the fermentation of dietary fiber and the secondary BA that are produced by bacterially catalyzed 7-hydroxylation reactions can effectively reduce inflammation. Moreover, activation of the tryptophan-AHR pathway is crucial for maintaining the normal functioning of the gut mucosal barrier and significantly reduces its dysfunction caused by pro-inflammatory factors. Additionally, a diet rich in dietary fiber plays a vital role in preventing inflammatory diseases. Therefore, focusing on these interactions can provide a potential for more in-depth investigations to unearth mechanisms underlying gut microbial-host interactions.

Although numerous studies have investigated the impact of gut microbial metabolites on host immunity, much of this relationship remains elusive. The precise mechanism by which SCFA promote the formation of TJ proteins and enhance the function of the intestinal epithelial barrier is still unknown. Additionally, the underlying mechanisms through which IPA improves impaired intestinal epithelial barrier function require further investigation. It is worth noting that certain studies have indicated that high concentrations of butyrate can be detrimental to the intestinal barrier, while low concentrations of butyrate are beneficial for its function. Consequently, it is essential to explore whether an excess of SCFA in the intestine can indeed prove harmful to the intestinal barrier, thus reconciling this apparent contradiction. Clarifying this aspect would necessitate additional research efforts.

## Author contributions

YF: Writing – original draft, Writing – review & editing. JL: Writing – original draft, Writing – review & editing. SW: Funding acquisition, Writing – review & editing.

## References

[1] Gu M Samuelson DR de la Rua NM Charles TP Taylor CM Luo M. Host innate and adaptive immunity shapes the gut microbiota biogeography. Microbiol Immunol (2022) 66(6):330–41. doi: 10.1111/1348-0421.12963 PMC9189012 35067963

[2] Weng YJ Gan HY Li X Huang Y Li ZC Deng HM. Correlation of diet, microbiota and metabolite networks in inflammatory bowel disease. J Dig Dis (2019) 20(9):447–59. doi: 10.1111/1751-2980.12795 31240835

[3] Rempel J Grover K El-Matary W. Micronutrient deficiencies and anemia in children with inflammatory bowel disease. Nutrients (2021) 13(1):236. doi: 10.3390/nu13010236 33467587 PMC7830649

[4] Sun Y Yuan S Chen X Sun J Kalla R Yu L. The contribution of genetic risk and lifestyle factors in the development of adult-onset inflammatory bowel disease: A prospective cohort study. Am J Gastroenterol (2023) 118(3):511–22. doi: 10.14309/ajg.0000000000002180 PMC9973435 36695739

[5] Kitamoto S Nagao-Kitamoto H Jiao Y Gillilland MG 3rd Hayashi A Imai J. The intermucosal connection between the mouth and gut in commensal pathobiont-driven colitis. Cell (2020) 182(2):447–62.e14. doi: 10.1016/j.cell.2020.05.048 32758418 PMC7414097

[6] Wu J Peters BA Dominianni C Zhang Y Pei Z Yang L. Cigarette smoking and the oral microbiome in a large study of American adults. Isme J (2016) 10(10):2435–46. doi: 10.1038/ismej.2016.37 PMC5030690 27015003

[7] Vogtmann E Flores R Yu G Freedman ND Shi J Gail MH. Association between tobacco use and the upper gastrointestinal microbiome among Chinese men. Cancer Causes Control. (2015) 26(4):581–8. doi: 10.1007/s10552-015-0535-2 PMC4852095 25701246

[8] Almradi A Hanzel J Sedano R Parker CE Feagan BG Ma C. Clinical trials of IL-12/IL-23 inhibitors in inflammatory bowel disease. BioDrugs (2020) 34(6):713–21. doi: 10.1007/s40259-020-00451-w 33105016

[9] Lacerda JF Lagos AC Carolino E Silva-Herdade AS Silva M Sousa Guerreiro C. Functional food components, intestinal permeability and inflammatory markers in patients with inflammatory bowel disease. Nutrients (2021) 13(2):642. doi: 10.3390/nu13020642 33669400 PMC7920414

[10] Parada Venegas D de la Fuente MK Landskron G González MJ Quera R Dijkstra G. Short chain fatty acids (SCFAs)-mediated gut epithelial and immune regulation and its relevance for inflammatory bowel diseases. Front Immunol (2019) 10:277. doi: 10.3389/fimmu.2019.00277 30915065 PMC6421268

[11] Zhou J Li M Chen Q Li X Chen L Dong Z. Programmable probiotics modulate inflammation and gut microbiota for inflammatory bowel disease treatment after effective oral delivery. Nat Commun (2022) 13(1):3432. doi: 10.1038/s41467-022-31171-0 35701435 PMC9198027

[12] Chu ND Crothers JW Nguyen LTT Kearney SM Smith MB Kassam Z. Dynamic colonization of microbes and their functions after fecal microbiota transplantation for inflammatory bowel disease. mBio (2021) 12(4):e0097521. doi: 10.1128/mBio.00975-21 34281401 PMC8406238

[13] Wang MX Lin L Chen YD Zhong YP Lin YX Li P. Evodiamine has therapeutic efficacy in ulcerative colitis by increasing Lactobacillus acidophilus levels and acetate production. Pharmacol Res (2020) 159:104978. doi: 10.1016/j.phrs.2020.104978 32485282

[14] Xu M Shen Y Cen M Zhu Y Cheng F Tang L. Modulation of the gut microbiota-farnesoid X receptor axis improves deoxycholic acid-induced intestinal inflammation in mice. J Crohns Colitis. (2021) 15(7):1197–210. doi: 10.1093/ecco-jcc/jjab003 33417675

[15] Michaudel C Danne C Agus A Magniez A Aucouturier A Spatz M. Rewiring the altered tryptophan metabolism as a novel therapeutic strategy in inflammatory bowel diseases. Gut (2023) 72(7):1296–307. doi: 10.1136/gutjnl-2022-327337 PMC10314090 36270778

[16] Martin KS Azzolini M Lira Ruas J. The kynurenine connection: how exercise shifts muscle tryptophan metabolism and affects energy homeostasis, the immune system, and the brain. Am J Physiol Cell Physiol (2020) 318(5):C818–c30. doi: 10.1152/ajpcell.00580.2019 32208989

[17] Lee JY Cevallos SA Byndloss MX Tiffany CR Olsan EE Butler BP. High-fat diet and antibiotics cooperatively impair mitochondrial bioenergetics to trigger dysbiosis that exacerbates pre-inflammatory bowel disease. Cell Host Microbe (2020) 28(2):273–84.e6. doi: 10.1016/j.chom.2020.06.001 32668218 PMC7429289

[18] Fan Y Pedersen O. Gut microbiota in human metabolic health and disease. Nat Rev Microbiol (2021) 19(1):55–71. doi: 10.1038/s41579-020-0433-9 32887946

[19] Lai S Yan Y Pu Y Lin S Qiu JG Jiang BH. Enterotypes of the human gut mycobiome. Microbiome (2023) 11(1):179. doi: 10.1186/s40168-023-01586-y 37563687 PMC10416509

[20] Fujio-Vejar S Vasquez Y Morales P Magne F Vera-Wolf P Ugalde JA. The gut microbiota of healthy Chilean subjects reveals a high abundance of the phylum verrucomicrobia. Front Microbiol (2017) 8:1221. doi: 10.3389/fmicb.2017.01221 28713349 PMC5491548

[21] Liu Y Yang M Tang L Wang F Huang S Liu S. TLR4 regulates RORγt(+) regulatory T-cell responses and susceptibility to colon inflammation through interaction with Akkermansia muciniphila. Microbiome (2022) 10(1):98. doi: 10.1186/s40168-022-01296-x 35761415 PMC9235089

[22] Van Averbeke V Berkell M Mysara M Rodriguez-Ruiz JP Xavier BB De Winter FHR. Host immunity influences the composition of murine gut microbiota. Front Immunol (2022) 13:828016. doi: 10.3389/fimmu.2022.828016 35371073 PMC8965567

[23] Vacca M Raspini B Calabrese FM Porri D De Giuseppe R Chieppa M. The establishment of the gut microbiota in 1-year-aged infants: from birth to family food. Eur J Nutr (2022) 61(5):2517–30. doi: 10.1007/s00394-022-02822-1 PMC9279275 35211851

[24] Grech A Collins CE Holmes A Lal R Duncanson K Taylor R. Maternal exposures and the infant gut microbiome: a systematic review with meta-analysis. Gut Microbes (2021) 13(1):1–30. doi: 10.1080/19490976.2021.1897210 PMC8276657 33978558

[25] de Vos WM Tilg H Van Hul M Cani PD. Gut microbiome and health: mechanistic insights. Gut (2022) 71(5):1020–32. doi: 10.1136/gutjnl-2021-326789 PMC8995832 35105664

[26] Beller L Deboutte W Falony G Vieira-Silva S Tito RY Valles-Colomer M. Successional stages in infant gut microbiota maturation. mBio (2021) 12(6):e0185721. doi: 10.1128/mbio.01857-21 34903050 PMC8686833

[27] Laursen MF Sakanaka M von Burg N Mörbe U Andersen D Moll JM. Bifidobacterium species associated with breastfeeding produce aromatic lactic acids in the infant gut. Nat Microbiol (2021) 6(11):1367–82. doi: 10.1038/s41564-021-00970-4 PMC8556157 34675385

[28] Brennan CA Clay SL Lavoie SL Bae S Lang JK Fonseca-Pereira D. Fusobacterium nucleatum drives a pro-inflammatory intestinal microenvironment through metabolite receptor-dependent modulation of IL-17 expression. Gut Microbes (2021) 13(1):1987780. doi: 10.1080/19490976.2021.1987780 34781821 PMC8604392

[29] Bolte LA Vich Vila A Imhann F Collij V Gacesa R Peters V. Long-term dietary patterns are associated with pro-inflammatory and anti-inflammatory features of the gut microbiome. Gut (2021) 70(7):1287–98. doi: 10.1136/gutjnl-2020-322670 PMC8223641 33811041

[30] Becattini S Sorbara MT Kim SG Littmann EL Dong Q Walsh G. Rapid transcriptional and metabolic adaptation of intestinal microbes to host immune activation. Cell Host Microbe (2021) 29(3):378–93.e5. doi: 10.1016/j.chom.2021.01.003 33539766 PMC7954923

[31] Larsen IS Jensen BAH Bonazzi E Choi BSY Kristensen NN Schmidt EGW. Fungal lysozyme leverages the gut microbiota to curb DSS-induced colitis. Gut Microbes (2021) 13(1):1988836. doi: 10.1080/19490976.2021.1988836 34693864 PMC8547870

[32] Wu H Xie S Miao J Li Y Wang Z Wang M. Lactobacillus reuteri maintains intestinal epithelial regeneration and repairs damaged intestinal mucosa. Gut Microbes (2020) 11(4):997–1014. doi: 10.1080/19490976.2020.1734423 32138622 PMC7524370

[33] Rangan P Choi I Wei M Navarrete G Guen E Brandhorst S. Fasting-mimicking diet modulates microbiota and promotes intestinal regeneration to reduce inflammatory bowel disease pathology. Cell Rep (2019) 26(10):2704–19.e6. doi: 10.1016/j.celrep.2019.02.019 30840892 PMC6528490

[34] Mihaylova MM Cheng CW Cao AQ Tripathi S Mana MD Bauer-Rowe KE. Fasting activates fatty acid oxidation to enhance intestinal stem cell function during homeostasis and aging. Cell Stem Cell (2018) 22(5):769–78.e4. doi: 10.1016/j.stem.2018.04.001 29727683 PMC5940005

[35] Bell HN Rebernick RJ Goyert J Singhal R Kuljanin M Kerk SA. Reuterin in the healthy gut microbiome suppresses colorectal cancer growth through altering redox balance. Cancer Cell (2022) 40(2):185–200.e6. doi: 10.1016/j.ccell.2021.12.001 34951957 PMC8847337

[36] Engevik MA Luk B Chang-Graham AL Hall A Herrmann B Ruan W. Bifidobacterium dentium Fortifies the Intestinal Mucus Layer via Autophagy and Calcium Signaling Pathways. mBio (2019) 10(3):e01087-19. doi: 10.1128/mBio.01087-19 31213556 PMC6581858

[37] Niu MM Guo HX Cai JW Meng XC. Bifidobacterium breve alleviates DSS-induced colitis in mice by maintaining the mucosal and epithelial barriers and modulating gut microbes. Nutrients (2022) 14(18):3671. doi: 10.3390/nu14183671 36145047 PMC9503522

[38] Fan L Qi Y Qu S Chen X Li A Hendi M. adolescentis ameliorates chronic colitis by regulating Treg/Th2 response and gut microbiota remodeling. Gut Microbes (2021) 13(1):1–17. doi: 10.1080/19490976.2020.1826746 PMC7889144 33557671

[39] Mowat AM. To respond or not to respond - a personal perspective of intestinal tolerance. Nat Rev Immunol (2018) 18(6):405–15. doi: 10.1038/s41577-018-0002-x 29491358

[40] Allam-Ndoul B Castonguay-Paradis S Veilleux A. Gut microbiota and intestinal trans-epithelial permeability. Int J Mol Sci (2020) 21(17):6402. doi: 10.3390/ijms21176402 32899147 PMC7503654

[41] Shan M Gentile M Yeiser JR Walland AC Bornstein VU Chen K. Mucus enhances gut homeostasis and oral tolerance by delivering immunoregulatory signals. Sci (New York NY). (2013) 342(6157):447–53. doi: 10.1126/science.1237910 PMC4005805 24072822

[42] Panpetch W Hiengrach P Nilgate S Tumwasorn S Somboonna N Wilantho A. Additional Candida albicans administration enhances the severity of dextran sulfate solution induced colitis mouse model through leaky gut-enhanced systemic inflammation and gut-dysbiosis but attenuated by Lactobacillus rhamnosus L34. Gut Microbes (2020) 11(3):465–80. doi: 10.1080/19490976.2019.1662712 PMC7527076 31530137

[43] Zegarra Ruiz DF Kim DV Norwood K Saldana-Morales FB Kim M Ng C. Microbiota manipulation to increase macrophage IL-10 improves colitis and limits colitis-associated colorectal cancer. Gut Microbes (2022) 14(1):2119054. doi: 10.1080/19490976.2022.2119054 36062329 PMC9450902

[44] Duan JL He HQ Yu Y Liu T Ma SJ Li F. E3 ligase c-Cbl regulates intestinal inflammation through suppressing fungi-induced noncanonical NF-κB activation. Sci Adv (2021) 7(19):eabe5171. doi: 10.1126/sciadv.abe5171 33962939 PMC8104877

[45] Doron I Mesko M Li XV Kusakabe T Leonardi I Shaw DG. Mycobiota-induced IgA antibodies regulate fungal commensalism in the gut and are dysregulated in Crohn’s disease. Nat Microbiol (2021) 6(12):1493–504. doi: 10.1038/s41564-021-00983-z PMC8622360 34811531

[46] Wang S Fu W Zhao X Chang X Liu H Zhou L. Zearalenone disturbs the reproductive-immune axis in pigs: the role of gut microbial metabolites. Microbiome (2022) 10(1):234. doi: 10.1186/s40168-021-01212-9 36536466 PMC9762105

[47] Wassie T Cheng B Zhou T Gao L Lu Z Xie C. Microbiome-metabolome analysis reveals alterations in the composition and metabolism of caecal microbiota and metabolites with dietary Enteromorpha polysaccharide and Yeast glycoprotein in chickens. Front Immunol (2022) 13:996897. doi: 10.3389/fimmu.2022.996897 36311785 PMC9614668

[48] Hold GL. Gastrointestinal microbiota and colon cancer. Dig Dis (2016) 34(3):244–50. doi: 10.1159/000443358 27028619

[49] Yang W Cong Y. Gut microbiota-derived metabolites in the regulation of host immune responses and immune-related inflammatory diseases. Cell Mol Immunol (2021) 18(4):866–77. doi: 10.1038/s41423-021-00661-4 PMC8115644 33707689

[50] Sanders ME Merenstein DJ Reid G Gibson GR Rastall RA. Probiotics and prebiotics in intestinal health and disease: from biology to the clinic. Nat Rev Gastroenterol hepatol (2019) 16(10):605–16. doi: 10.1038/s41575-019-0173-3 31296969

[51] Alfa MJ Strang D Tappia PS Graham M Van Domselaar G Forbes JD. A randomized trial to determine the impact of a digestion resistant starch composition on the gut microbiome in older and mid-age adults. Clin Nutr (2018) 37(3):797–807. doi: 10.1016/j.clnu.2017.03.025 28410921

[52] Gasaly N de Vos P Hermoso MA. Impact of bacterial metabolites on gut barrier function and host immunity: A focus on bacterial metabolism and its relevance for intestinal inflammation. Front Immunol (2021) 12:658354. doi: 10.3389/fimmu.2021.658354 34122415 PMC8187770

[53] Makki K Deehan EC Walter J Bäckhed F. The impact of dietary fiber on gut microbiota in host health and disease. Cell Host Microbe (2018) 23(6):705–15. doi: 10.1016/j.chom.2018.05.012 29902436

[54] Reichardt N Vollmer M Holtrop G Farquharson FM Wefers D Bunzel M. Specific substrate-driven changes in human faecal microbiota composition contrast with functional redundancy in short-chain fatty acid production. Isme J (2018) 12(2):610–22. doi: 10.1038/ismej.2017.196 PMC5776475 29192904

[55] Mu Q Kirby J Reilly CM Luo XM. Leaky gut as a danger signal for autoimmune diseases. Front Immunol (2017) 8:598. doi: 10.3389/fimmu.2017.00598 28588585 PMC5440529

[56] Beukema M Faas MM de Vos P. The effects of different dietary fiber pectin structures on the gastrointestinal immune barrier: impact via gut microbiota and direct effects on immune cells. Exp Mol Med (2020) 52(9):1364–76. doi: 10.1038/s12276-020-0449-2 PMC8080816 32908213

[57] Al Bander Z Nitert MD Mousa A Naderpoor N. The gut microbiota and inflammation: an overview. Int J Environ Res Public Health (2020) 17(20):7618. doi: 10.3390/ijerph17207618 33086688 PMC7589951

[58] Zhao J Zhang X Liu H Brown MA Qiao S. Dietary protein and gut microbiota composition and function. Curr Protein Pept sci (2019) 20(2):145–54. doi: 10.2174/1389203719666180514145437 29756574

[59] Sun M Wu W Liu Z Cong Y. Microbiota metabolite short chain fatty acids, GPCR, and inflammatory bowel diseases. J Gastroenterol (2017) 52(1):1–8. doi: 10.1007/s00535-016-1242-9 27448578 PMC5215992

[60] Michael OS Dibia CL Soetan OA Adeyanju OA Oyewole AL Badmus OO. Sodium acetate prevents nicotine-induced cardiorenal dysmetabolism through uric acid/creatine kinase-dependent pathway. Life Sci (2020) 257:118127. doi: 10.1016/j.lfs.2020.118127 32707052

[61] Sugita K Kabashima K. Tight junctions in the development of asthma, chronic rhinosinusitis, atopic dermatitis, eosinophilic esophagitis, and inflammatory bowel diseases. J Leukoc Biol (2020) 107(5):749–62. doi: 10.1002/JLB.5MR0120-230R 32108379

[62] Peng L Li ZR Green RS Holzman IR Lin J. Butyrate enhances the intestinal barrier by facilitating tight junction assembly *via* activation of AMP-activated protein kinase in Caco-2 cell monolayers. J Nutr (2009) 139(9):1619–25. doi: 10.3945/jn.109.104638 PMC2728689 19625695

[63] Xia W Khan I Li XA Huang G Yu Z Leong WK. Adaptogenic flower buds exert cancer preventive effects by enhancing the SCFA-producers, strengthening the epithelial tight junction complex and immune responses. Pharmacol Res (2020) 159:104809. doi: 10.1016/j.phrs.2020.104809 32502642

[64] Martin-Gallausiaux C Marinelli L Blottière HM Larraufie P Lapaque N. SCFA: mechanisms and functional importance in the gut. Proc Nutr Soc (2021) 80(1):37–49. doi: 10.1017/S0029665120006916 32238208

[65] Kellow NJ Coughlan MT Reid CM. Metabolic benefits of dietary prebiotics in human subjects: a systematic review of randomised controlled trials. Br J Nutr (2014) 111(7):1147–61. doi: 10.1017/S0007114513003607 24230488

[66] Carretta MD Quiroga J López R Hidalgo MA Burgos RA. Participation of short-chain fatty acids and their receptors in gut inflammation and colon cancer. Front Physiol (2021) 12:662739. doi: 10.3389/fphys.2021.662739 33897470 PMC8060628

[67] Thangaraju M Cresci GA Liu K Ananth S Gnanaprakasam JP Browning DD. GPR109A is a G-protein-coupled receptor for the bacterial fermentation product butyrate and functions as a tumor suppressor in colon. Cancer Res (2009) 69(7):2826–32. doi: 10.1158/0008-5472.CAN-08-4466 PMC3747834 19276343

[68] Thibault R De Coppet P Daly K Bourreille A Cuff M Bonnet C. Down-regulation of the monocarboxylate transporter 1 is involved in butyrate deficiency during intestinal inflammation. Gastroenterology (2007) 133(6):1916–27. doi: 10.1053/j.gastro.2007.08.041 18054563

[69] Nancey S Blanvillain E Parmentier B Flourié B Bayet C Bienvenu J. Infliximab treatment does not induce organ-specific or nonorgan-specific autoantibodies other than antinuclear and anti-double-stranded DNA autoantibodies in Crohn’s disease. Inflammation Bowel Dis (2005) 11(11):986–91. doi: 10.1097/01.MIB.0000186408.07769.78 16239844

[70] Mangan MSJ Olhava EJ Roush WR Seidel HM Glick GD Latz E. Targeting the NLRP3 inflammasome in inflammatory diseases. Nat Rev Drug Discovery (2018) 17(8):588–606. doi: 10.1038/nrd.2018.97 30026524

[71] Schlatterer K Peschel A Kretschmer D. Short-chain fatty acid and FFAR2 activation - A new option for treating infections? Front Cell Infect Microbiol (2021) 11:785833. doi: 10.3389/fcimb.2021.785833 34926327 PMC8674814

[72] Kespohl M Vachharajani N Luu M Harb H Pautz S Wolff S. The microbial metabolite butyrate induces expression of th1-associated factors in CD4(+) T cells. Front Immunol (2017) 8:1036. doi: 10.3389/fimmu.2017.01036 28894447 PMC5581317

[73] Serino M. Molecular paths linking metabolic diseases, gut microbiota dysbiosis and enterobacteria infections. J Mol Biol (2018) 430(5):581–90. doi: 10.1016/j.jmb.2018.01.010 29374557

[74] Jang HR Lee HY. Mechanisms linking gut microbial metabolites to insulin resistance. World J diabetes. (2021) 12(6):730–44. doi: 10.4239/wjd.v12.i6.730 PMC8192250 34168724

[75] Vinolo MA Rodrigues HG Nachbar RT Curi R. Regulation of inflammation by short chain fatty acids. Nutrients (2011) 3(10):858–76. doi: 10.3390/nu3100858 PMC3257741 22254083

[76] Nafday SM Chen W Peng L Babyatsky MW Holzman IR Lin J. Short-chain fatty acids induce colonic mucosal injury in rats with various postnatal ages. Pediatr Res (2005) 57(2):201–4. doi: 10.1203/01.PDR.0000150721.83224.89 15611351

[77] Peng L He Z Chen W Holzman IR Lin J. Effects of butyrate on intestinal barrier function in a Caco-2 cell monolayer model of intestinal barrier. Pediatr Res (2007) 61(1):37–41. doi: 10.1203/01.pdr.0000250014.92242.f3 17211138

[78] Donohoe DR Collins LB Wali A Bigler R Sun W Bultman SJ. The Warburg effect dictates the mechanism of butyrate-mediated histone acetylation and cell proliferation. Mol Cell (2012) 48(4):612–26. doi: 10.1016/j.molcel.2012.08.033 PMC3513569 23063526

[79] Sivaprakasam S Gurav A Paschall AV Coe GL Chaudhary K Cai Y. An essential role of Ffar2 (Gpr43) in dietary fibre-mediated promotion of healthy composition of gut microbiota and suppression of intestinal carcinogenesis. Oncogenesis (2016) 5(6):e238. doi: 10.1038/oncsis.2016.38 27348268 PMC4945739

[80] Macfarlane S Macfarlane GT. Regulation of short-chain fatty acid production. Proc Nutr Soc (2003) 62(1):67–72. doi: 10.1079/PNS2002207 12740060

[81] Cross TL Kasahara K Rey FE. Sexual dimorphism of cardiometabolic dysfunction: Gut microbiome in the play? Mol Metab (2018) 15:70–81. doi: 10.1016/j.molmet.2018.05.016 29887245 PMC6066746

[82] Dawson PA Lan T Rao A. Bile acid transporters. J Lipid Res (2009) 50(12):2340–57. doi: 10.1194/jlr.R900012-JLR200 PMC2781307 19498215

[83] Kanaley JA Colberg SR Corcoran MH Malin SK Rodriguez NR Crespo CJ. Exercise/physical activity in individuals with type 2 diabetes: A consensus statement from the american college of sports medicine. Med Sci Sports Exerc. (2022) 54(2):353–68. doi: 10.1249/MSS.0000000000002800 PMC8802999 35029593

[84] Ridlon JM Kang DJ Hylemon PB. Isolation and characterization of a bile acid inducible 7alpha-dehydroxylating operon in Clostridium hylemonae TN271. Anaerobe (2010) 16(2):137–46. doi: 10.1016/j.anaerobe.2009.05.004 PMC6262846 19464381

[85] Chiang JY. Bile acids: regulation of synthesis. J Lipid Res (2009) 50(10):1955–66. doi: 10.1194/jlr.R900010-JLR200 PMC2739756 19346330

[86] Fiorucci S Carino A Baldoni M Santucci L Costanzi E Graziosi L. Bile acid signaling in inflammatory bowel diseases. Dig Dis Sci (2021) 66(3):674–93. doi: 10.1007/s10620-020-06715-3 PMC7935738 33289902

[87] Withers DR Hepworth MR Wang X Mackley EC Halford EE Dutton EE. Transient inhibition of ROR-γt therapeutically limits intestinal inflammation by reducing TH17 cells and preserving group 3 innate lymphoid cells. Nat Med (2016) 22(3):319–23. doi: 10.1038/nm.4046 PMC4948756 26878233

[88] Ocvirk S O’Keefe SJD. Dietary fat, bile acid metabolism and colorectal cancer. Semin Cancer Biol (2021) 73:347–55. doi: 10.1016/j.semcancer.2020.10.003 33069873

[89] Sun L Xie C Wang G Wu Y Wu Q Wang X. Gut microbiota and intestinal FXR mediate the clinical benefits of metformin. Nat Med (2018) 24(12):1919–29. doi: 10.1038/s41591-018-0222-4 PMC6479226 30397356

[90] Fiorucci S Distrutti E Carino A Zampella A Biagioli M. Bile acids and their receptors in metabolic disorders. Prog Lipid Res (2021) 82:101094. doi: 10.1016/j.plipres.2021.101094 33636214

[91] Monte MJ Marin JJ Antelo A Vazquez-Tato J. Bile acids: chemistry, physiology, and pathophysiology. World J Gastroenterol (2009) 15(7):804–16. doi: 10.3748/wjg.15.804 PMC2653380 19230041

[92] de Aguiar Vallim TQ Tarling EJ Edwards PA. Pleiotropic roles of bile acids in metabolism. Cell Metab (2013) 17(5):657–69. doi: 10.1016/j.cmet.2013.03.013 PMC3654004 23602448

[93] Sayin SI Wahlström A Felin J Jäntti S Marschall HU Bamberg K. Gut microbiota regulates bile acid metabolism by reducing the levels of tauro-beta-muricholic acid, a naturally occurring FXR antagonist. Cell Metab (2013) 17(2):225–35. doi: 10.1016/j.cmet.2013.01.003 23395169

[94] Long SL Gahan CGM Joyce SA. Interactions between gut bacteria and bile in health and disease. Mol Aspects Med (2017) 56:54–65. doi: 10.1016/j.mam.2017.06.002 28602676

[95] Jones BV Begley M Hill C Gahan CG Marchesi JR. Functional and comparative metagenomic analysis of bile salt hydrolase activity in the human gut microbiome. Proc Natl Acad Sci U S A. (2008) 105(36):13580–5. doi: 10.1073/pnas.0804437105 PMC2533232 18757757

[96] Kundu S Kumar S Bajaj A. Cross-talk between bile acids and gastrointestinal tract for progression and development of cancer and its therapeutic implications. IUBMB Life (2015) 67(7):514–23. doi: 10.1002/iub.1399 26177921

[97] Fiorucci S Biagioli M Zampella A Distrutti E. Bile acids activated receptors regulate innate immunity. Front Immunol (2018) 9:1853. doi: 10.3389/fimmu.2018.01853 30150987 PMC6099188

[98] Cipriani S Mencarelli A Chini MG Distrutti E Renga B Bifulco G. The bile acid receptor GPBAR-1 (TGR5) modulates integrity of intestinal barrier and immune response to experimental colitis. PloS One (2011) 6(10):e25637. doi: 10.1371/journal.pone.0025637 22046243 PMC3203117

[99] Fiorucci S Distrutti E. Bile acid-activated receptors, intestinal microbiota, and the treatment of metabolic disorders. Trends Mol Med (2015) 21(11):702–14. doi: 10.1016/j.molmed.2015.09.001 26481828

[100] Yang M Gu Y Li L Liu T Song X Sun Y. Bile acid-gut microbiota axis in inflammatory bowel disease: from bench to bedside. Nutrients (2021) 13(9):3143. doi: 10.3390/nu13093143 34579027 PMC8467364

[101] Lu TT Makishima M Repa JJ Schoonjans K Kerr TA Auwerx J. Molecular basis for feedback regulation of bile acid synthesis by nuclear receptors. Mol Cell (2000) 6(3):507–15. doi: 10.1016/S1097-2765(00)00050-2 11030331

[102] Cariello M Piccinin E Garcia-Irigoyen O Sabbà C Moschetta A Nuclear receptor FXR. bile acids and liver damage: Introducing the progressive familial intrahepatic cholestasis with FXR mutations. Biochim Biophys Acta Mol Basis Dis (2018) 1864(4 Pt B):1308–18. doi: 10.1016/j.bbadis.2017.09.019 28965883

[103] Chávez-Talavera O Tailleux A Lefebvre P Staels B. Bile acid control of metabolism and inflammation in obesity, type 2 diabetes, dyslipidemia, and nonalcoholic fatty liver disease. Gastroenterology (2017) 152(7):1679–94.e3. doi: 10.1053/j.gastro.2017.01.055 28214524

[104] Liu JZ van Sommeren S Huang H Ng SC Alberts R Takahashi A. Association analyses identify 38 susceptibility loci for inflammatory bowel disease and highlight shared genetic risk across populations. Nat Genet (2015) 47(9):979–86. doi: 10.1038/ng.3359 PMC4881818 26192919

[105] Heinken A Ravcheev DA Baldini F Heirendt L Fleming RMT Thiele I. Systematic assessment of secondary bile acid metabolism in gut microbes reveals distinct metabolic capabilities in inflammatory bowel disease. Microbiome (2019) 7(1):75. doi: 10.1186/s40168-019-0689-3 31092280 PMC6521386

[106] Sinha SR Haileselassie Y Nguyen LP Tropini C Wang M Becker LS. Dysbiosis-induced secondary bile acid deficiency promotes intestinal inflammation. Cell Host Microbe (2020) 27(4):659–70.e5. doi: 10.1016/j.chom.2020.01.021 32101703 PMC8172352

[107] Wang L Gong Z Zhang X Zhu F Liu Y Jin C. Gut microbial bile acid metabolite skews macrophage polarization and contributes to high-fat diet-induced colonic inflammation. Gut Microbes (2020) 12(1):1–20. doi: 10.1080/19490976.2020.1819155 PMC7553752 33006494

[108] Carr RM Reid AE. FXR agonists as therapeutic agents for non-alcoholic fatty liver disease. Curr Atheroscl Rep (2015) 17(4):500. doi: 10.1007/s11883-015-0500-2 25690590

[109] Jia W Xie G Jia W. Bile acid-microbiota crosstalk in gastrointestinal inflammation and carcinogenesis. Nat Rev Gastroenterol hepatol (2018) 15(2):111–28. doi: 10.1038/nrgastro.2017.119 PMC5899973 29018272

[110] Jia ET Liu ZY Pan M Lu JF Ge QY. Regulation of bile acid metabolism-related signaling pathways by gut microbiota in diseases. J Zhejiang Univ Sci B (2019) 20(10):781–92. doi: 10.1631/jzus.B1900073 PMC6751489 31489798

[111] Sun R Xu C Feng B Gao X Liu Z. Critical roles of bile acids in regulating intestinal mucosal immune responses. Therap Adv Gastroenterol (2021) 14:17562848211018098. doi: 10.1177/17562848211018098 PMC8165529 34104213

[112] Ala M. Tryptophan metabolites modulate inflammatory bowel disease and colorectal cancer by affecting immune system. Int Rev Immunol (2022) 41(3):326–45. doi: 10.1080/08830185.2021.1954638 34289794

[113] Fiore A Murray PJ. Tryptophan and indole metabolism in immune regulation. Curr Opin Immunol (2021) 70:7–14. doi: 10.1016/j.coi.2020.12.001 33418116

[114] Cherayil BJ. Indoleamine 2,3-dioxygenase in intestinal immunity and inflammation. Inflammation Bowel Dis (2009) 15(9):1391–6. doi: 10.1002/ibd.20910 19322906

[115] Campbell BM Charych E Lee AW Möller T. Kynurenines in CNS disease: regulation by inflammatory cytokines. Front Neurosci (2014) 8:12. doi: 10.3389/fnins.2014.00012 24567701 PMC3915289

[116] Chung DJ Rossi M Romano E Ghith J Yuan J Munn DH. Indoleamine 2,3-dioxygenase-expressing mature human monocyte-derived dendritic cells expand potent autologous regulatory T cells. Blood (2009) 114(3):555–63. doi: 10.1182/blood-2008-11-191197 PMC2713474 19465693

[117] Wang SJ Sharkey KA McKay DM. Modulation of the immune response by helminths: a role for serotonin? Biosci Rep (2018) 38(5):BSR20180027. doi: 10.1042/BSR20180027 PMC6148219 30177522

[118] Yano JM Yu K Donaldson GP Shastri GG Ann P Ma L. Indigenous bacteria from the gut microbiota regulate host serotonin biosynthesis. Cell (2015) 161(2):264–76. doi: 10.1016/j.cell.2015.02.047 PMC4393509 25860609

[119] Alexeev EE Lanis JM Kao DJ Campbell EL Kelly CJ Battista KD. Microbiota-derived indole metabolites promote human and murine intestinal homeostasis through regulation of interleukin-10 receptor. Am J Pathol (2018) 188(5):1183–94. doi: 10.1016/j.ajpath.2018.01.011 PMC5906738 29454749

[120] Hills RD Jr. Pontefract BA Mishcon HR Black CA Sutton SC Theberge CR. Gut microbiome: profound implications for diet and disease. Nutrients (2019) 11(7):1613. doi: 10.3390/nu11071613 31315227 PMC6682904

[121] Haghikia A Zimmermann F Schumann P Jasina A Roessler J Schmidt D. Propionate attenuates atherosclerosis by immune-dependent regulation of intestinal cholesterol metabolism. Eur Heart J (2022) 43(6):518–33. doi: 10.1093/eurheartj/ehab644 PMC9097250 34597388

[122] Brandsma E Kloosterhuis NJ Koster M Dekker DC Gijbels MJJ van der Velden S. A proinflammatory gut microbiota increases systemic inflammation and accelerates atherosclerosis. Circ Res (2019) 124(1):94–100. doi: 10.1161/CIRCRESAHA.118.313234 30582442 PMC6325767

[123] Yang W Yu T Huang X Bilotta AJ Xu L Lu Y. Intestinal microbiota-derived short-chain fatty acids regulation of immune cell IL-22 production and gut immunity. Nat Commun (2020) 11(1):4457. doi: 10.1038/s41467-020-18262-6 32901017 PMC7478978

[124] Li G Lin J Zhang C Gao H Lu H Gao X. Microbiota metabolite butyrate constrains neutrophil functions and ameliorates mucosal inflammation in inflammatory bowel disease. Gut Microbes (2021) 13(1):1968257. doi: 10.1080/19490976.2021.1968257 34494943 PMC8437544

[125] Li L Zhang Y Speakman JR Hu S Song Y Qin S. The gut microbiota and its products: Establishing causal relationships with obesity related outcomes. Obes reviews: an Off J Int Assoc Study Obes (2021) 22(12):e13341. doi: 10.1111/obr.13341 34490704

[126] Caesar R. Pharmacologic and nonpharmacologic therapies for the gut microbiota in type 2 diabetes. Can J diabetes. (2019) 43(3):224–31. doi: 10.1016/j.jcjd.2019.01.007 30929665

[127] Sehgal R de Mello VD Mannisto V Lindstrom J Tuomilehto J Pihlajamaki J. Indolepropionic acid, a gut bacteria-produced tryptophan metabolite and the risk of type 2 diabetes and non-alcoholic fatty liver disease. Nutrients (2022) 14(21):4695. doi: 10.3390/nu14214695 36364957 PMC9653718

[128] Qi Q Li J Yu B Moon JY Chai JC Merino J. Host and gut microbial tryptophan metabolism and type 2 diabetes: an integrative analysis of host genetics, diet, gut microbiome and circulating metabolites in cohort studies. Gut (2022) 71(6):1095–105. doi: 10.1136/gutjnl-2021-324053 PMC8697256 34127525

[129] Shen J Yang L You K Chen T Su Z Cui Z. Indole-3-acetic acid alters intestinal microbiota and alleviates ankylosing spondylitis in mice. Front Immunol (2022) 13:762580. doi: 10.3389/fimmu.2022.762580 35185872 PMC8854167

[130] Ji Y Gao Y Chen H Yin Y Zhang W. Indole-3-acetic acid alleviates nonalcoholic fatty liver disease in mice via attenuation of hepatic lipogenesis, and oxidative and inflammatory stress. Nutrients (2019) 11(9):2062. doi: 10.3390/nu11092062 31484323 PMC6769627

[131] Li Y Zhou M Li C Pan X Lv N Ye Z. Inoculating indoleacetic acid bacteria promotes the enrichment of halotolerant bacteria during secondary fermentation of composting. J Environ Manage (2022) 322:116021. doi: 10.1016/j.jenvman.2022.116021 36067675

[132] Neinast M Murashige D Arany Z. Branched chain amino acids. Annu Rev Physiol (2019) 81:139–64. doi: 10.1146/annurev-physiol-020518-114455 PMC6536377 30485760

[133] Le Couteur DG Solon-Biet SM Cogger VC Ribeiro R de Cabo R Raubenheimer D. Branched chain amino acids, aging and age-related health. Ageing Res Rev (2020) 64:101198. doi: 10.1016/j.arr.2020.101198 33132154

[134] Schoeler M Caesar R. Dietary lipids, gut microbiota and lipid metabolism. Rev endocrine Metab Disord (2019) 20(4):461–72. doi: 10.1007/s11154-019-09512-0 PMC6938793 31707624

[135] Krautkramer KA Fan J Bäckhed F. Gut microbial metabolites as multi-kingdom intermediates. Nat Rev Microbiol (2021) 19(2):77–94. doi: 10.1038/s41579-020-0438-4 32968241

[136] Correia AS Vale N. Tryptophan metabolism in depression: A narrative review with a focus on serotonin and kynurenine pathways. Int J Mol Sci (2022) 23(15):8493. doi: 10.3390/ijms23158493 35955633 PMC9369076

[137] van Galen KA Ter Horst KW Serlie MJ. Serotonin, food intake, and obesity. Obes reviews: an Off J Int Assoc Study Obes (2021) 22(7):e13210. doi: 10.1111/obr.13210 PMC8243944 33559362

[138] Huda MN Kim M Bennett BJ. Modulating the microbiota as a therapeutic intervention for type 2 diabetes. Front Endocrinol (2021) 12:632335. doi: 10.3389/fendo.2021.632335 PMC8060771 33897618

[139] Roager HM Licht TR. Microbial tryptophan catabolites in health and disease. Nat Commun (2018) 9(1):3294. doi: 10.1038/s41467-018-05470-4 30120222 PMC6098093

[140] Keszthelyi D Troost FJ Masclee AA. Understanding the role of tryptophan and serotonin metabolism in gastrointestinal function. Neurogastroenterol Motil. (2009) 21(12):1239–49. doi: 10.1111/j.1365-2982.2009.01370.x 19650771

[141] Zhai L Wu J Lam YY Kwan HY Bian ZX Wong HLX. Gut-microbial metabolites, probiotics and their roles in type 2 diabetes. Int J Mol Sci (2021) 22(23):12846. doi: 10.3390/ijms222312846 34884651 PMC8658018

[142] Hashimoto T Perlot T Rehman A Trichereau J Ishiguro H Paolino M. ACE2 links amino acid malnutrition to microbial ecology and intestinal inflammation. Nature (2012) 487(7408):477–81. doi: 10.1038/nature11228 PMC7095315 22837003

[143] Gutiérrez-Vázquez C Quintana FJ. Regulation of the immune response by the aryl hydrocarbon receptor. Immunity (2018) 48(1):19–33. doi: 10.1016/j.immuni.2017.12.012 29343438 PMC5777317

[144] Su X Gao Y Yang R. Gut microbiota-derived tryptophan metabolites maintain gut and systemic homeostasis. Cells (2022) 11(15):2296. doi: 10.3390/cells11152296 35892593 PMC9330295

[145] Sun M Ma N He T Johnston LJ Ma X. Tryptophan (Trp) modulates gut homeostasis via aryl hydrocarbon receptor (AhR). Crit Rev Food Sci Nutr (2020) 60(10):1760–8. doi: 10.1080/10408398.2019.1598334 30924357

[146] Hubbard TD Murray IA Bisson WH Sullivan AP Sebastian A Perry GH. Divergent ah receptor ligand selectivity during hominin evolution. Mol Biol Evol (2016) 33(10):2648–58. doi: 10.1093/molbev/msw143 PMC5026259 27486223

[147] Hidaka T Ogawa E Kobayashi EH Suzuki T Funayama R Nagashima T. The aryl hydrocarbon receptor AhR links atopic dermatitis and air pollution via induction of the neurotrophic factor artemin. Nat Immunol (2017) 18(1):64–73. doi: 10.1038/ni.3614 27869817

[148] Mescher M Haarmann-Stemmann T. Modulation of CYP1A1 metabolism: From adverse health effects to chemoprevention and therapeutic options. Pharmacol Ther (2018) 187:71–87. doi: 10.1016/j.pharmthera.2018.02.012 29458109

[149] Lamas B Hernandez-Galan L Galipeau HJ Constante M Clarizio A Jury J. Aryl hydrocarbon receptor ligand production by the gut microbiota is decreased in celiac disease leading to intestinal inflammation. Sci Transl Med (2020) 12(566):eaba0624. doi: 10.1126/scitranslmed.aba0624 33087499

[150] Park SL Justiniano R Williams JD Cabello CM Qiao S Wondrak GT. The tryptophan-derived endogenous aryl hydrocarbon receptor ligand 6-formylindolo[3,2-b]Carbazole is a nanomolar UVA photosensitizer in epidermal keratinocytes. J Invest Dermatol (2015) 135(6):1649–58. doi: 10.1038/jid.2014.503 PMC4430374 25431849

[151] Wang F Graham WV Wang Y Witkowski ED Schwarz BT Turner JR. Interferon-gamma and tumor necrosis factor-alpha synergize to induce intestinal epithelial barrier dysfunction by up-regulating myosin light chain kinase expression. Am J Pathol (2005) 166(2):409–19. doi: 10.1016/S0002-9440(10)62264-X PMC1237049 15681825

[152] Yamada T Horimoto H Kameyama T Hayakawa S Yamato H Dazai M. Constitutive aryl hydrocarbon receptor signaling constrains type I interferon-mediated antiviral innate defense. Nat Immunol (2016) 17(6):687–94. doi: 10.1038/ni.3422 27089381

[153] Jennis M Cavanaugh CR Leo GC Mabus JR Lenhard J Hornby PJ. Microbiota-derived tryptophan indoles increase after gastric bypass surgery and reduce intestinal permeability in *vitro* and in vivo. Neurogastroenterol Motil. (2018) 30(2). doi: 10.1111/nmo.13178 28782205

[154] Wlodarska M Luo C Kolde R d’Hennezel E Annand JW Heim CE. Indoleacrylic acid produced by commensal peptostreptococcus species suppresses inflammation. Cell Host Microbe (2017) 22(1):25–37.e6. doi: 10.1016/j.chom.2017.06.007 28704649 PMC5672633

[155] Whitfield-Cargile CM Cohen ND Chapkin RS Weeks BR Davidson LA Goldsby JS. The microbiota-derived metabolite indole decreases mucosal inflammation and injury in a murine model of NSAID enteropathy. Gut Microbes (2016) 7(3):246–61. doi: 10.1080/19490976.2016.1156827 PMC4939928 27007819

[156] Lamas B Richard ML Sokol H. Caspase recruitment domain 9, microbiota, and tryptophan metabolism: dangerous liaisons in inflammatory bowel diseases. Curr Opin Clin Nutr Metab Care (2017) 20(4):243–7. doi: 10.1097/MCO.0000000000000382 28399013

